# Quantitative assessment of lipophilic membrane dye‐based labelling of extracellular vesicles by nano‐flow cytometry

**DOI:** 10.1002/jev2.12351

**Published:** 2023-07-31

**Authors:** Chen Chen, Niangui Cai, Qian Niu, Ye Tian, Yunyun Hu, Xiaomei Yan

**Affiliations:** ^1^ Department of Chemical Biology, MOE Key Laboratory of Spectrochemical Analysis & Instrumentation, Key Laboratory for Chemical Biology of Fujian Province, State Key Laboratory of Physical Chemistry of Solid Surfaces, College of Chemistry and Chemical Engineering Xiamen University Xiamen People's Republic of China

**Keywords:** extracellular vesicles, lipophilic membrane dyes, lipophilic membrane probes, membrane labelling, nano‐flow cytometry, single particle analysis

## Abstract

Although lipophilic membrane dyes (LMDs) or probes (LMPs) are widely used to label extracellular vesicles (EVs) for detection and purification, their labelling performance has not been systematically characterized. Through concurrent side scattering and fluorescence detection of single EVs as small as 40 nm in diameter by a laboratory‐built nano‐flow cytometer (nFCM), present study identified that (1) PKH67 and PKH26 could maximally label ∼60%–80% of EVs isolated from the conditioned cell culture medium (purity of ∼88%) and ∼40%–70% of PFP‐EVs (purity of ∼73%); (2) excessive PKH26 could cause damage to the EV structure; (3) di‐8‐ANEPPS and high concentration of DiI could achieve efficient and uniform labelling of EVs with nearly 100% labelling efficiency for di‐8‐ANEPPS and 70%–100% for DiI; (4) all the four tested LMDs can aggregate and form micelles that exhibit comparable side scatter and fluorescence intensity with those of labelled EVs and thus hardly be differentiate from each other; (5) as the LMD concentration went up, the particle number of self‐aggregates increased while the fluorescence intensity of aggregates remained constant; (6) PKH67 and PKH26 tend to form more aggregated micelles than di‐8‐ANEPPS and DiI, and the effect of LMD self‐aggregation can be negligible at optimal staining conditions. (7) All the four tested LMDs can label almost all the very‐low‐density lipoprotein (VLDL) particles, indicating potential confounding factor in plasma‐EV labelling. Besides, it was discovered that DSPE‐PEG_2000_‐biotin can only label ∼50% of plasma‐EVs. The number of LMP inserted into the membrane of single EVs was measured for the first time and it was confirmed that membrane labelling by lipophilic dyes did not interfere with the immunophenotyping of EVs. nFCM provides a unique perspective for a better understanding of EV labelling by LMD/LMP.

## 1 INTRODUCTION

Extracellular vesicles (EVs) have attracted much attention owing to their critical roles in pathophysiological processes, as well as their great clinical potential as disease biomarkers and vectors for drug delivery (Herrmann et al., 2021; Melo et al., 2015; van Niel et al., 2018; Xu et al., 2018). EVs isolated from cell lines or body fluids constitute a heterogeneous population of vesicles with different sizes, secretion pathways, and molecular compositions (Kalluri, 2020; Willms et al., 2018; Zijlstra et al., 2018). Since the vast majority of EVs are smaller than 200 nm as measured by cryo‐transmission electron microscopy (TEM) (Tian et al., 2018; Zabeo et al., 2017) and there lacks a universal EV protein marker (Thery et al., 2018), it has been extremely challenging to study EVs on a single‐particle basis. Nevertheless, the double‐layered lipid membrane represents the most prominent characteristics of EVs, and fluorescent labelling with lipophilic membrane dyes (LMDs) (e.g., PKH dyes and Di‐Dyes) has been widely used to visualize and detect individual EVs by fluorescent microscopy and flow cytometry (F. Chen et al., 2019; Choi et al., 2019; Grange et al., 2014; Kamerkar et al., 2017; van der Vlist et al., 2012; Yong et al., 2019). Moreover, fluorescent membrane staining has been used for counting and sizing EVs by flow cytometry, and the kits are commercially available from Cellarcus Biosciences and in wide use (Oh et al., 2022; Park et al., 2022; Sandau et al., 2020; Shpigelman et al., 2021; Yan et al., 2022). Besides, lipid membrane probes (LMPs) have also been applied for EV capture and enrichment from complex matrix via the insertion of lipid‐anchored probes into the EV membrane (Di et al., 2019; Sun et al., 2022; Wan et al., 2017).

Despite the increasing use of LMDs for EV monitoring, there are generally three major pitfalls associated with LMD‐based EV labelling. First, can LMDs label all the EVs? Fluorescence nanoparticle tracking analysis (NTA) and highly sensitive flow cytometric analysis indicated that the EV labelling efficiency of LMDs is not high (de Rond et al., 2018; Kamei et al., 2021; Midekessa et al., 2021). Second, LMDs tend to self‐aggregate and form micelles that resemble the size, structure, and fluorescence intensity of EVs (Dehghani et al., 2020; Dominkus et al., 2018; Lai et al., 2015; Morales‐Kastresana et al., 2017; Simonsen, 2019), which could lead to false positive signals. Third, will non‐EV particles be concomitantly labelled? There exists a considerable overlap in particle size and density of EVs and lipoproteins and the ∼four to six orders of magnitude higher concentration of lipoproteins than EVs in human plasma usually lead to unintentional coisolation of lipoproteins with EVs from plasma (Karimi et al., 2018; Simonsen, 2017; Tian et al., 2020). Lipoproteins, covered by a lipid monolayer on their surface, could also be labelled by LMDs (Mathieu et al., 2019; Takov et al., 2017). Quantitative assessment of these staining issues is crucial to ensure the validity of any conclusions drawn for EVs that rely on LMD‐fluorescence.

As every EV particle scatters light, elastic light scattering represents the simplest and most direct method of EV particle detection because it is label‐free. Once the scattered light of each individual EVs can be distinguished from the background, it can be used as a hallmark of a particle event (that covers all the EVs) (Lian et al., 2019). For an EV preparation with high purity, the EV labelling ratio of LMDs can be accurately measured by comparing the number of fluorescent EVs with the number of total EVs. However, the efficiency of EV detection by light scatter depends on the size and refractive index of the EV and the sensitivity of the instrument. NTA has difficulty detecting EVs smaller than 70−100 nm based on light scattering (Gardiner et al., 2014; van der Pol, Coumans, Sturk, et al., 2014), and the minimum detectable EV size is around 200 nm via light scattering by the high‐end conventional flow cytometers (van der Pol, de Rond, et al., 2018; van der Pol, Sturk, et al., 2018). Employing strategies for single molecule fluorescence detection in a sheathed flow, our laboratory developed nano‐flow cytometer (nFCM) that is capable of light scattering detection of single silica nanoparticles and EVs as small as 24 and 40 nm, respectively (Tian et al., 2018; Zhu et al., 2014). The sizing resolution and accuracy of EVs are comparable to that of cryo‐TEM (Tian et al., 2022, 2018) and the fluorescence detection limit was three Alexa Fluor 532 molecules (Zhu et al., 2014). The superior sensitivity of nFCM in the concurrent light scattering and fluorescence measurement of single EVs provides us a unique opportunity to quantitatively evaluate the performance of LMDs in EV labelling with unprecedented detail and precision. In the present study, side scatter triggering was applied as a reference, and the labelling efficiency of EVs, degree of self‐aggregation, and staining of lipoproteins were investigated for four most commonly used LMDs, PKH67, PKH26, di‐8‐ANEPPS, and DiI. EVs isolated from the cultured cells and platelet‐free plasma (PFP) were studied. The effectiveness of DSPE‐PEG_2000_‐biotin for EV capture and the influence on immunophenotyping were also examined.

## 2 MATERIALS AND METHODS

### 2.1 Cell culture

The human colorectal cancer cell line (HCT‐15) and a normal colon fibroblast cell line (CCD‐18Co) were purchased from the American Type Culture Collection (ATCC). HCT‐15 cells were cultured in RPMI 1640 medium (Gibco, 11875‐093), and CCD‐18Co cells were cultured in minimal essential medium (MEM) (Gibco, 41500‐067). All media were supplemented with 10% FBS (ExCell Bio, FSP‐500, Uruguay) and penicillin‐streptomycin (Invitrogen). The FBS used above was depleted of EVs by ultracentrifugation at 100,000 × g for 18 h at 4°C (Beckman Coulter X‐90 centrifuge, SW41 Ti rotor). Cells were grown in EV‐depleted medium until they reached a confluence of ∼90% (after approximately 24 h) before the culture medium was collected.

### 2.2 Isolation of EVs from cell culture conditioned medium and plasma

The cell culture media was centrifuged at 800 × g for 5 min at 4°C to pellet the cells. The supernatant was centrifuged at 2,000 × g for 10 min at 4°C to remove cellular debris and is called the conditioned cell culture medium (CCCM) in the present study. Freshly prepared CCCM (12.5 mL) or thawed PFP (2.0 mL) diluted to 12.5 mL with PBS were centrifuged at 100,000 × g for 2 h at 4°C (Beckman Coulter XE‐90K Ultracentrifuge, SW 41 Ti rotor). The pellet was washed with 12.5 mL of PBS and followed by a second ultracentrifugation (UC) at 100,000 × g for 2 h at 4°C. Afterwards, the supernatant was discarded and EVs were resuspended in 50–100 μL PBS.

### 2.3 Lipid extraction for untargeted lipidomics analysis

To a 200 μL of EV sample with a concentration of approximately 5 × 10^11^/mL (measured by nFCM), 200 μL cold water, 20 μL lipid internal standard mixture from Avanti Polar Lipids, Inc., and 800 μL cold methyl tert‐butyl ether were added and vortexed for 30 s. Then, 240 μL methanol was added. The mixture was vortexed for 30 s, sonicated at 4°C for 20 min, stood at room temperature for 30 min, and then centrifuged at 14,000 g for 15 min at 10°C to extract lipids. The upper organic layer was dried in a vacuum centrifuge, and the lipid extract was resuspended in 200 μL of isopropanol acetonitrile 9:1 (v/v). For untargeted lipidomics analysis, the extract was analyzed by LC‐MS by Shanghai Applied Protein Technology Co., Ltd (See Supplementary Information for details).

### 2.4 Synthesis of liposomes

Liposomes were prepared by film hydration and extrusion as described in the literature (C. X. Chen et al., 2017). Briefly, DSPC (1,2‐Dioctadecanoyl‐sn‐glycero‐3‐phophocholine) and cholesterol (purchased from Avanti Polar Lipids, Inc.) were mixed and dissolved in chloroform at a molar ratio of 60:40 with a total lipid concentration of 100 mM. Once the lipids were thoroughly mixed, the solvent was dried on a rotary evaporator to form a lipid film and kept in vacuo for at least 3 h to remove the residual chloroform. The lipid film was hydrated in PBS solution at 65°C for 1 h followed by sequential extrusion through polycarbonate membranes with pore size of 400, 200, 100, 80, and 50 nm to obtain homogeneous particle size. After 20 cycles of extrusion with every size of the polycarbonate filters down to 50 nm, the synthesized liposomes were stored at 4°C for further use.

### 2.5 Lipophilic membrane dye labelling

PKH67, PKH26, Diluent C, and DiI were purchased from Sigma‐Aldrich. Pluronic F‐127 and di‐8‐ANEPPS were purchased from Invitrogen. All the dyes were dissolved in DMSO and stored at 4°C for further use. Purified VLDL (American Research Products, 12−4020, purity >95%, 1.48 mg/mL) was filtered through a 0.22‐μm filter for further use. EV samples, liposomes, and VLDL were diluted to approximately 1.5 × 10^10^ particles/mL (measure by nFCM) in Diluent C (for PKH67 and PKH26 labelling) or PBS (for di‐8‐ANEPPS and DiI labelling). The guidelines provided by the manufacturer and literature were followed (van der Vlist et al., 2012). For labelling with PKH dyes, 50 μL EV sample was pipetted into 50 μL PKH67 or PKH26 solution and incubated for 3 min at room temperature. Then 100 μL PKH stop solution (RPMI1640 cell condition media with 10% vesicle‐free FBS) was added to the mixture and incubated for 1 min to stop the staining reaction. For di‐8‐ANEPPS labelling, 50 μL EV sample was added to 50 μL di‐8‐ANEPPS solution containing 0.1% Pluronic F‐127 and incubated at 4°C for 20 min. For DiI labelling, 50 μL EV sample was added to 50 μL DiI solution and incubated for 10 min at room temperature. The final labelling concentrations of LMDs were 1, 2, 4, and 8 μM, respectively. Reagent controls were carried out by adding corresponding buffer instead of EVs for each concentration of the four LMDs, respectively. To remove free LMDs, the mixture was washed twice with 1 mL PBS by ultracentrifugation at 100,000 × g for 17 min at 4°C (Beckman Coulter MAX‐XP centrifuge, TLA‐120.2 rotor). The pellet was resuspended in 50 μL PBS for nFCM analysis. Noting that centrifuging at 100,000 × g for 17 min with Beckman Coulter Optima Max‐XP ultracentrifuge equipped with a TLA 120.2 rotor offers the same fractionation effect as that of centrifuging at 100,000 × g for 120 min with Beckman Coulter XE‐90K Ultracentrifuge equipped with an SW41 Ti rotor.

### 2.6 Lipophilic membrane probe (LMP) labelling

EV samples or VLDL were diluted to 1.5 × 10^10^ particles/mL in Diluent C. Then 50 μL EV sample was added to 50 μL DSPE‐PEG_2000_‐biotin (Avanti Polar Lipids, Inc.) pre‐diluted with Diluent C and incubated for 10 min at room temperature. The final labelling concentrations of DSPE‐PEG_2000_‐biotin were 1, 2, 4, and 8 μM, respectively. Negative controls were carried out by adding corresponding buffer instead of EVs for each concentration of DSPE‐PEG_2000_‐biotin. The mixture was washed with 1 mL PBS by ultracentrifugation at 100,000 × g for 17 min at 4°C (Beckman Coulter MAX‐XP centrifuge, TLA‐120.2 rotor) to remove free LMP molecules. The pellet was resuspended in 100 μL PBS. Then 1 μL of 2 mg/mL streptavidin‐Alexa Flour 532 (Thermo Scientific) was added and incubated for 20 min at 37°C. The mixture was washed twice with 1 mL PBS by ultracentrifugation at 100,000 × g for 17 min at 4°C (Beckman Coulter MAX‐XP centrifuge, TLA‐120.2 rotor). The pellet was resuspended in 50 μL PBS for nFCM analysis.

### 2.7 nFCM analysis

The laboratory‐built nFCM reported before was used in the present study (Tian et al., 2018; Zhu et al., 2014). Briefly, two single‐photon counting avalanche photodiodes (APDs) were used for the simultaneous detection of the SSC (FF01 – 488/6 bandpass filter for a 488 nm laser or a FF01 – 524/24 bandpass filter for a 532 nm laser) and FL (FF01 – 525/45 bandpass filter for green fluorescence, FF01 – 579/34 bandpass filter for orange fluorescence, or FF01 – 630/69 bandpass filter for red fluorescence) of individual EVs, respectively. Unless stated otherwise, each distribution histogram or dot‐plot was derived from data collected 1 min. Ultrapure water supplied by an ultrapure water system (PURELAB Ultra FLC00006307, ELGA) served as the sheath fluid via gravity feed. On the basis of the overlap of the focused laser spot and the sample stream, the calculated detection volume was about 25 fL. According to Poisson statistics, for a nanoparticle concentration of approximately 5 × 10^9^ particles/mL, the probability that two nanoparticles will pass through the detection volume simultaneously is 0.7%. For nFCM analysis, the sample stream is completely illuminated within the central region of the focused laser beam, and the detection efficiency is approximately 100%, which leads to accurate particle concentration measurement via single particle enumeration (Yang et al., 2009; Zhu et al., 2010). The concentration of EV samples, liposomes, and VLDL was determined by employing 100 nm orange FluoSpheres (Thermo Fisher, F8803) of known particle concentration to calibrate the sample flow rate. Several dilutions were made to the orange FluoSpheres solution, and a linear correlation between particle concentration and detected event rate was obtained with R^2^ of 0.999 (data not shown).

For measuring the particle size of EV preparations, a mixture of monodisperse silica nanoparticles (SiNPs) of five different diameters ranging from 47 to 123 nm (47, 59, 74, 95, and 123 nm) was analyzed under the same instrument conditions as those of EV analysis. By linearly interpolating the refractive indices of SiNPs at a wavelength nearby, the refractive indices of SiNPs at 532 nm were calculated to be 1.461 (Lide, 2010). Considering the refractive index difference between the SiNPs (1.461) and EVs (1.400) at 532 nm excitation, the intensity ratio between light scattered by a SiNP to that of an EV of the same particle size was calculated based on the Mie theory for every size of the SiNP standard (Tian et al., 2018; van der Pol, Coumans, Grootemaat, et al., 2014). These ratios were used as the correction factors to derive the calibration curve between the scatted light intensity and particle size of EVs from the data of SiNPs (Tian et al., 2022, 2018). The purity of EVs isolated from CCCM or PFP were measured as the detergent sensitivity by using nFCM to enumerate single nanoparticles via SSC detection and comparing the event rates detected in 1 min before and after Triton X‐100 treatment (Tian et al., 2022, 2018). Noting that the particle counts detected in 1 min for PBS buffer (∼250 event) was deducted from these event rates before the ratio calculation. Briefly, 10 μL of 10% Triton X‐100 (Sigma‐Aldrich, X100) was added to 90 μL of EV preparation with a particle concentration of approximately 6 × 10^9^ particles/mL. After 30 min of incubation at 4°C, the treated sample was diluted 20‐fold prior to the nFCM analysis.

### 2.8 Immunofluorescent staining of EVs

PE‐conjugated mouse anti‐human CD9 antibody (clone M‐L13, eBioscience), PE‐conjugated mouse anti‐human CD63 antibody (clone H5C6, BD Biosciences), PE‐conjugated mouse anti‐human CD81 antibody (clone JS‐81, BD Biosciences), and PE‐conjugated mouse IgG1, κ (clone MOCP‐21, BD Biosciences) were used in the present study. For immunofluorescent staining of EVs derived by HCT‐15 cells, purified EVs labelled with or without 8 μM DSPE‐PEG_2000_‐biotin were resuspended in 50 μL PBS. Into each 50 μL EV sample, 5 μL PE‐conjugated antibody against CD9, or 20 μL PE‐conjugated antibody against CD63, CD81, or IgG1 was added according to the manufacturer's guidelines. The mixture was incubated at 37°C for 30 min and then washed twice with 1 mL PBS by ultracentrifugation at 100,000 × g for 17 min at 4°C (Beckman Coulter MAX‐XP centrifuge, TLA‐120.2 rotor). The pellet was resuspended in 50 μL PBS for nFCM analysis.

## 3 RESULTS

### 3.1 Principle and characteristics of LMDs for lipid membrane labelling

**FIGURE 1:**
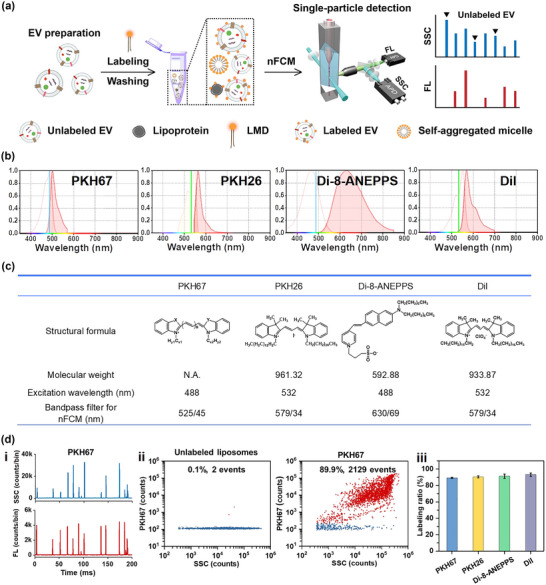
Principle of nFCM analysis and characteristics of LMDs for lipid membrane labelling. (a) A schematic diagram shows the work flow of EV membrane labelling by LMD along with nFCM analysis. (b) The fluorescence excitation and emission spectra of PKH67, PKH26, di‐8‐ANEPPS, and DiI. (c) Structure formula, molecular weight, and nFCM setup for these four LMDs. (d) Representative SSC and FL burst traces of a DSPC liposome sample stained by 4 μM PKH67 (i) and the bivariate dot‐plots of PKH67 fluorescence versus SSC for the unlabelled and PKH67 labelled liposome samples (ii), and the labelling ratios of DSPC liposomes stained by all four LMDs (iii).

Figure 1a illustrates the schematic diagram of EV membrane labelling by LMD along with single‐particle analysis by nFCM. The diluent of EV sample was added to the diluent of LMD of equal volume for a homogeneous labelling. Upon incorporating the long aliphatic tails into the lipid regions of the EV membrane, free LMDs in solution are removed from the LMD‐labelled EVs by washing. For an EV preparation with high purity, if fluorescence signal was not detected concurrently with SSC signal for a single EV, it can be recognized as unlabelled EVs. Thus, the percentage of fluorescent particles out of the total population (SSC detected) can be used to refer to the LMD labelling ratio of EVs. However, for an EV preparation with contaminants, the presence of fluorescent non‐EV particles or scattering non‐EV particles will interfere with the labelling ratio measurement which needs to be kept in mind. PKH67 (490/502 nm), PKH26 (551/567 nm), di‐8‐ANEPPS (465/635 nm), and DiI (549/565 nm), the four most commonly used LMDs for EV labelling were examined in the present study. Characteristics of their excitation spectrum, emission spectrum, structural formula, molecular weight, along with the excitation laser wavelength and the bandpass filter used for fluorescence detection on nFCM are provided in Figure 1b,c. In order to eliminate the interference from lipoproteins or other contaminants co‐isolated with EVs, synthetic liposomes can serve as an ideal model to assess the performance of LMDs on lipid‐membrane labelling (Stoner et al., 2016). Figure S1a shows the SSC burst area distribution histograms of unstained DSPC liposomes and PBS. Representative TEM micrographs of the DSPC liposome sample are displayed in Figure S1b. Figure 1d (i) shows the representative SSC and FL burst traces of a DSPC liposome sample stained by 4 μM PKH67. Clearly, concomitant FL and SSC signals were detected for almost all the particles. The bivariate dot‐plot of PKH67 fluorescence versus SSC shown in Figure 1d (ii) indicates that approximately 90% of the nanoparticles detectable by nFCM via SSC were fluorescently labelled by PKH67. The labelling ratio defined as the percentage of fluorescently positive population out of the total population is 90%. By using nFCM to enumerate single nanoparticles via SSC detection and comparing the event rates detected in 1 min before and after 1% Triton X‐100 treatment (Tian et al., 2022, 2018), the purity (defined as the detergent sensitivity) of the DSPC liposome sample was measured to be 93.2%. The influence of Triton X‐100 concentration on the purity determination of lipid membrane vesicles was investigated by measuring the event rate of PBS containing different volume fractions of Triton X‐100 without the presence of liposomes or EVs. Figure S2 reveals that the event rate detected in 1 min for Triton X‐100 solutions with volume fraction lower than 0.1% was comparable to that of the PBS control (∼250 events). When the volume fraction of Triton X‐100 reached 0.5%, a much higher event rate (∼750 events) was detected, confirming the formation of micelles which could affect the accuracy for purity determination. As the liposome or EV sample was diluted 20‐fold prior to the nFCM analysis upon 1% Triton X‐100 treatment at 4°C for 30 min, the final volume fraction of Triton X‐100 was 0.05% which would be compatible for purity assessment. Figure 1d (ii, iii) and Figure S1c,d show the labelling ratio of DSPC liposomes for all the four LMDs. The well‐matching of the liposome labelling ratios with the liposome purity indicates that these four LMDs can label nearly all the liposomes.

### 3.2 Characterization of EVs isolated from the CCCM of HCT‐15 cells

**FIGURE 2:**
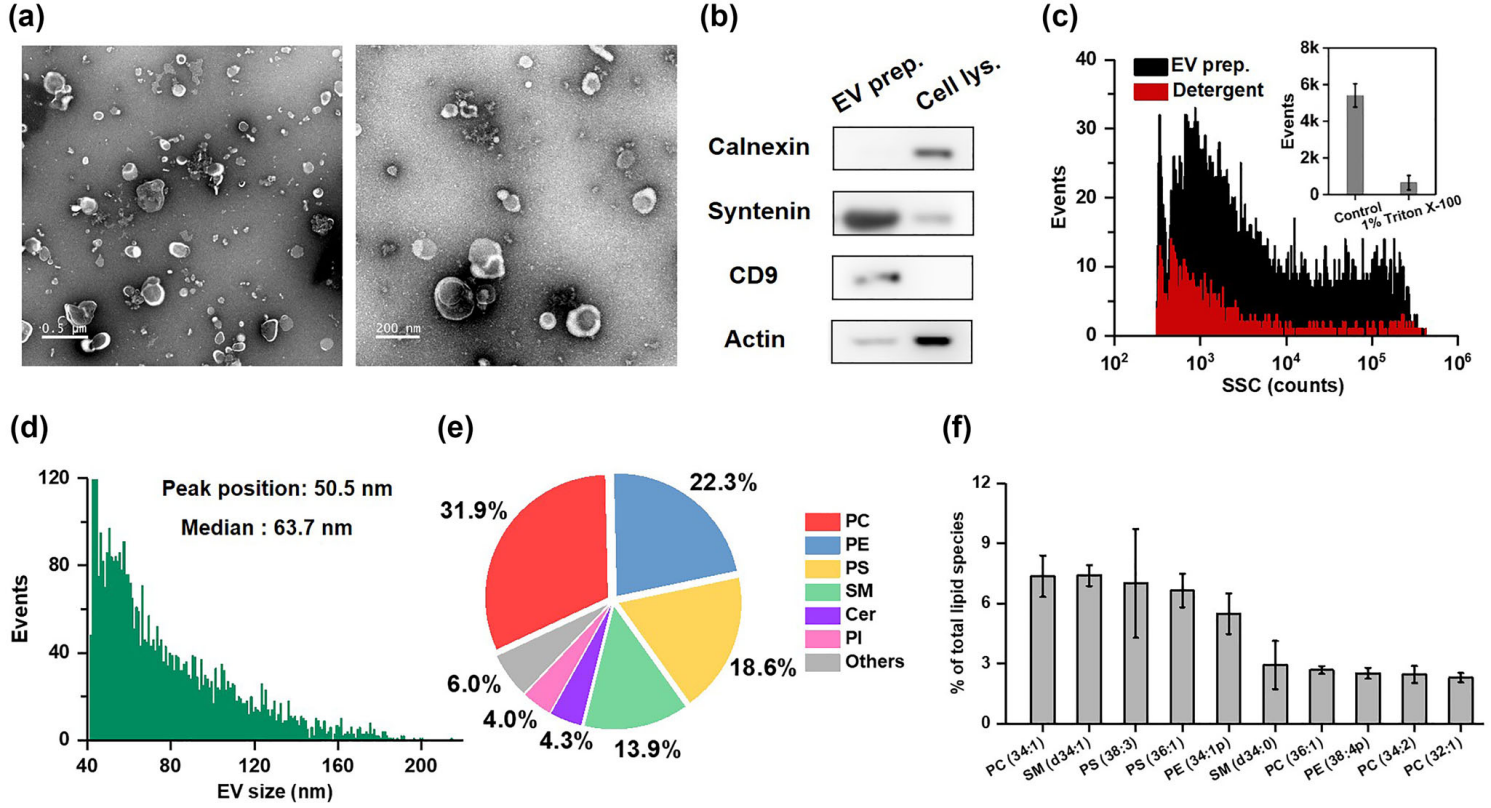
Characterization of EVs isolated from the CCCM of HCT‐15 cells by ultracentrifugation. (a) Representative TEM micrographs of an EV isolate, the scale bars are 500 and 200 nm, respectively. (b) Immunoblots of a cell lysate and an EV preparation. (c) SSC distribution histograms for an EV preparation before (black line) and after Triton X‐100 treatment (red line), along with the bar graph of event rate detected in 1 min for both samples (inset). The error bar represents the standard deviation (s.d.) of three replicate experiments. (d) Histogram of particle size with a bin width of 1 nm for an EV sample isolated from the CCCM of HCT‐15 cells by ultracentrifugation. (e) Pie chart representing the distribution of detected lipid classes of phosphatidylcholine (PC), phosphatidylethanolamine (PE), phosphatidylserine (PS), sphingomyelin (SM), ceramide (Cer), phosphatidylinositol (PI), and the rest lipid classes in EVs. The area of each pie chart is proportional to the corresponding lipid contents. (f) Lipid content for different lipid species was assessed for EVs of three different batches. Results are expressed as the mean ± SD. The ten lipid species with the highest contents are listed.

EVs isolated from the CCCM of HCT‐15 by ultracentrifugation served as model EV samples to examine EV labelling efficiency of LMDs. TEM and Western blotting results of the EV preparation are shown in Figure 2a,b. Figure 2c shows the SSC burst area distribution histograms of the EV sample before and after Triton X‐100 treatment. The event rate counted in 1 min dropped from 5606 to 521 upon 1% Triton X‐100 treatment and the purity (detergent sensitivity) of the EV sample was calculated to be 88.3% (*n* = 3). Figure 2d depicts the particle size distribution histogram of the EV sample by converting the SSC intensity of single EVs to particle size via the calibration curve constructed by monodisperse SiNPs along with refractive index mismatch correction (Figure S3). The EVs mainly fell in the size range of 40–200 nm, and the peak position and median size of EVS were measured to be 50.5 and 63.7 nm, respectively. To characterize the lipid membrane composition of EVs, lipidomics analysis was conducted by HPLC coupled with mass spectrometry (*n* = 3). A total of 23 lipid classes and 729 lipid species were found in the EV preparation of HCT‐15 cells. Figure 2e shows that the six most abundant lipid classes were phosphatidylcholines (PC, 31.9%), phosphatidylethanolamines (PE, 22.3%), phosphatidylserines (PS, 18.6%), sphingomyelins (SM, 13.9%), ceramide (Cer, 4.3%), and phosphatidylinositol (PI, 4.0%), which were similar to the previous reports (Hussey et al., 2020; Zhang et al., 2018). Meanwhile, the most abundant phospholipids were identified to be PC (34:1), SM (34:1), PS (38:3), and PS (36:1) (Figure 2f). Figure S4 shows the lipid content of less‐abundant lipid classes in the EV preparation of HCT‐15 cells. The lower content of TAG (triacylglycerol) and CL (cardiolipin) indicates that the level of lipoprotein and mitochondria contamination was low (Skotland et al., 2020). In order to evaluate the labelling efficiency of LMDs for PE and PS classes, DSPE and DMPS liposomes were synthesized following the protocol used for DSPC liposome synthesis. Figure S5 indicates that for all the four LMDs, the labelling ratios of DSPE and DMPS liposomes were all around ∼90%, exhibiting comparable performance with that of DSPC liposomes. Therefore, LMDs showed no difference in labelling PC, PE, and PS classes.

### 3.3 LMD labelling of EVs isolated from cultured cells

**FIGURE 3:**
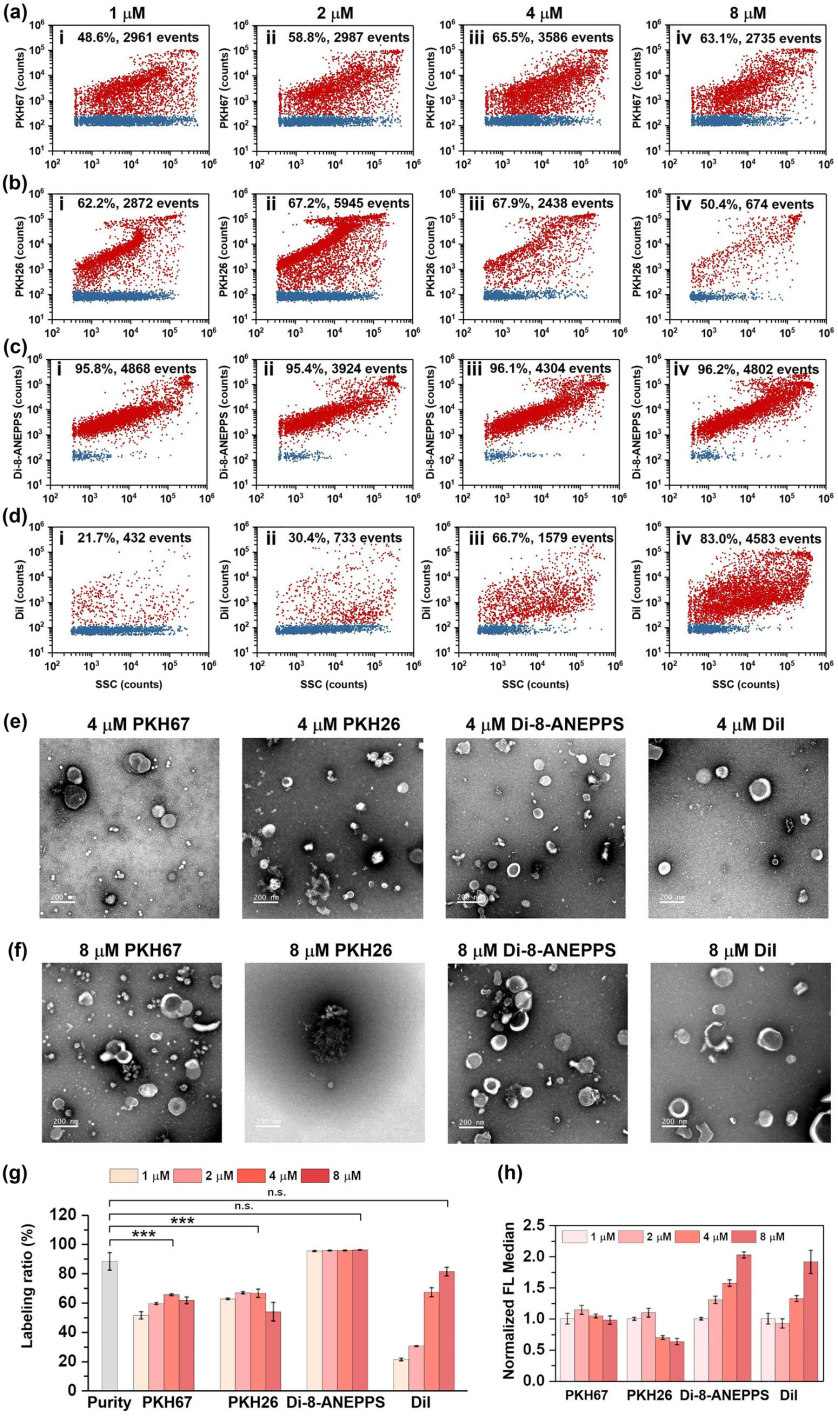
Analysis of membrane labelling efficiency of LMDs for EVs isolated from the CCCM of HCT‐15 cell line by ultracentrifugation. (a‐d) Bivariate dot‐plots of LMD fluorescence versus SSC for PKH67 (a), PKH26 (b), di‐8‐ANEPPS (c), and DiI (d)‐labelled EVs at 0 μM (i), 1 μM (ii), 2 μM (iii), 4 μM (iv), and 8 μM (v), respectively. (e and f) Typical TEM images of EVs labelled with PKH67, PKH26, di‐8‐ANEPPS, and DiI at 4 μM (e) and 8 μM (f). The scale bar is 200 nm. (g) Purity of EVs and labelling ratios for EVs stained with four different LMDs at four different concentrations (mean ± s.d.). (h) Normalized median FL intensity of EVs labelled by LMDs (*n* = 3, mean ± s.d.). Group differences between EV purity and the highest labelling ratio of different LMDs (4 μM for PKH67, 4 μM for PKH26, 8 μM for di‐8‐ANEPPS, and 8 μM for DiI) were tested by one‐way ANOVA analysis followed by post‐hoc Bonferroni's test for multiple comparison. *p*‐Value of <0.05 was considered statistically significant. *p*<0.05, *; *p*<0.01, **; *p*<0.001, ***.

To study the membrane labelling efficiency, EVs isolated from the CCCM of HCT‐15 cells by ultracentrifugation were diluted to 1.5 × 10^10^ particles/mL and labelled without or with different LMDs at 1 μM, 2 μM, 4 μM, and 8 μM, respectively. Figure 3a‐d shows the bivariate dot‐plots of LMD fluorescence versus SSC for PKH67, PKH26, Di‐8‐ANEPPS, and DiI, respectively. For PKH67 and PKH26, the highest labelling ratios were 65.5% and 67.9%, respectively, all obtained at 4 μM. Compared to the 88.3% purity of EVs (Figure 2c), it is clear that not all the EVs were fluorescently labelled with PKH dye even under saturating labelling conditions. Further increase of PKH concentration to 8 μM led to a decrease in the labelling ratio. In particular, a significant drop in the event rate was observed for PKH26‐stained EV sample at higher concentrations, which could be ascribed to the potential destruction of EV structure. By contrast, di‐8‐ANEPPS labelled all the EVs at all four dye concentrations with labelling ratios all higher than 95% (Figure 3c). For DiI, the labelling ratio of EVs increased steadily with the increase of dye concentration from 21.7% at 1 μM to 83.0% at 8 μM, and further increase to 12 μM and 16 μM did not enhance the labelling ratio (Figure 3d and Figure S6), implicating the importance of DiI concentration optimization for EV labelling.

To investigate whether high concentration of LMD labelling could alter the rigidity of the membrane and results in damage of EV integrity, TEM examination was conducted for EVs labelled with 4 μM and 8 μM of LMDs and the representative micrographs are shown in Figures 3e,f. For PKH67 labelling, despite the comparable structure with that of the unlabelled EVs (Figure 2a), some white dots were observed which could be ascribed to the self‐aggregation of PKH67. For EVs labelled with 4 μM of PKH26, broken EVs with a vague membrane structure were observed. When the dye concentration was increased to 8 μM, the number of EVs was scarce in the view of TEM images and agglomeration was clearly observed. Taking together the data of nFCM (Figure 3b), it is confirmed that excessive insertion of PKH26 into EV membrane could cause membrane damage that ultimately led to breakage of EVs. As the debris of EVs was removed upon washing, a decreased particle counts was observed on nFCM. Although it has been noted in the datasheet provided by the manufacture that over‐labelling of PKH dyes will result in loss of membrane integrity and reduce the recovery, nFCM analysis provides a quantitative assessment of EV damage upon excessive PKH labelling. Except for PKH26, EVs labelled with PKH67, di‐8‐ANEPPS, and DiI maintained intact membrane structure at all the tested LMD concentrations.

For an easy comparison, the labelling ratios and normalized median fluorescence intensity for all LMDs at four different concentrations are plotted in Figure 3g,h. Three replicates were conducted for each experiment. For PKH67 and PKH26, relatively higher FL intensity was obtained at 2 μM, and further increase of the dye concentration resulted in a decrease of FL intensity, which could be attributed to aggregation‐induced quenching caused by the saturation of membrane‐inserting PKH dyes (Ding et al., 2013). For di‐8‐ANEPPS and DiI, a continued increase of FL intensity with the increase of dye concentration was observed. The different behaviour of concentration‐dependent FL performance between PKH dyes and di‐8‐ANEPPS and DiI could be ascribed to their different head groups and hydrophobic chains (Faller, 2016; Osella et al., 2021). The LMD labelling results for EVs isolated from CCD‐18Co, a normal colon fibroblast cell line is displayed in Figure S7. Comparable results with HCT‐15 EVs were obtained except that slightly higher labelling ratios were obtained for PKH dyes.

### 3.4 LMD self‐aggregation assessment

**FIGURE 4:**
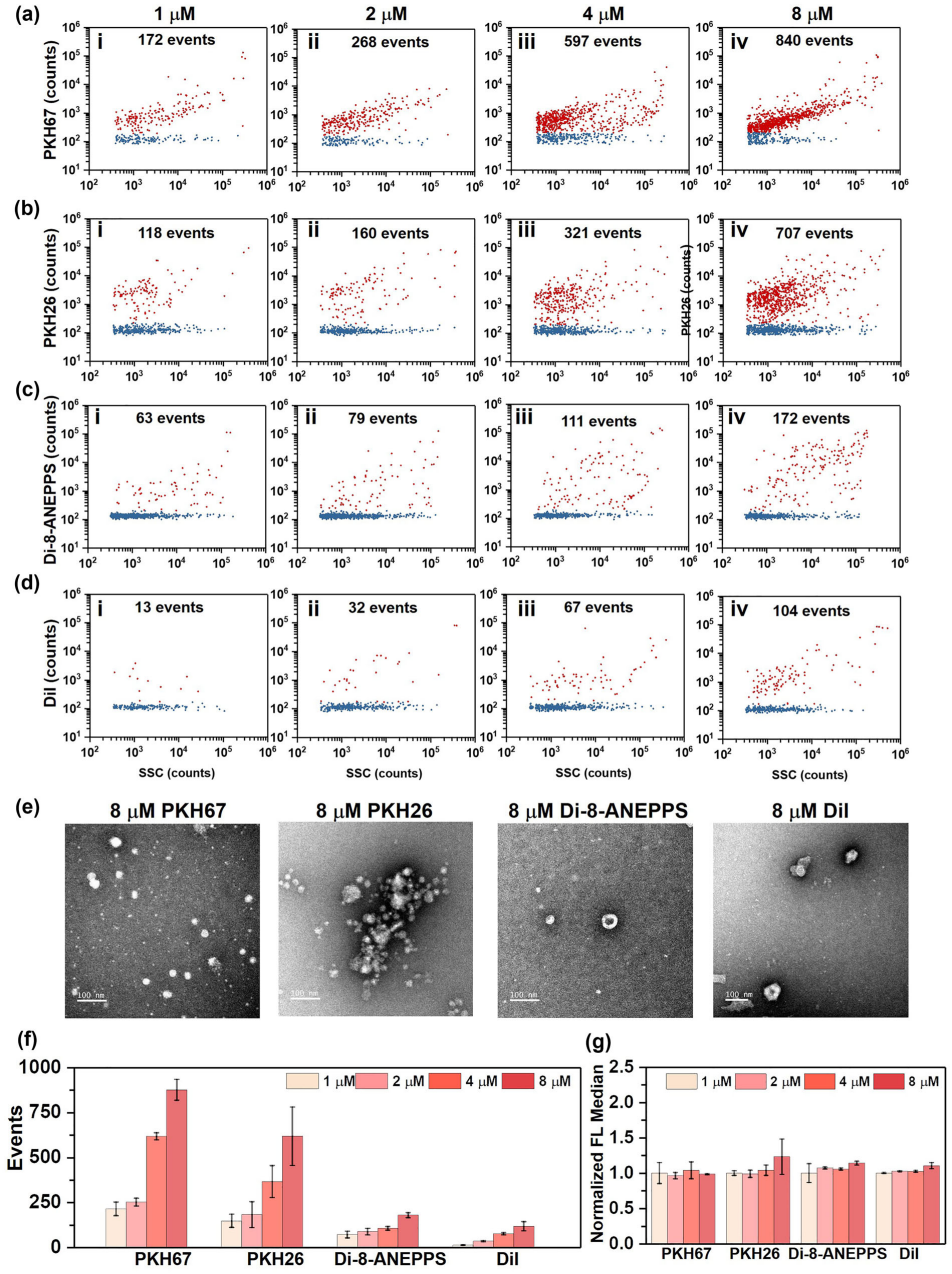
Self‐aggregation assessment of PKH67, PKH26, di‐8‐ANEPPS, and DiI. (a‐d) Bivariate dot‐plots of LMD fluorescence versus SSC for self‐aggregated micelles formed by PKH67 (a), PKH26 (b), di‐8‐ANEPPS (c), and DiI (d) at 1 μM (i), 2 μM (ii), 4 μM (iii), and 8 μM (iv), respectively. (e) Representative TEM images of aggregates formed at 8 μM LMD concentration for each dye. The scale bar is 200 nm. (f) Event rates of fluorescent self‐aggregates detected in 1 min for four LMDs at four different dye concentrations (*n* = 3, mean ± s.d.). (g) Normalized median FL intensity of self‐aggregates of PKH67, PKH26, di‐8‐ANEPPS and DiI at different concentrations (*n* = 3, mean ± s.d.).

Several studies have highlighted that LMDs tend to self‐aggregate and form micelles with similar size and morphology to those of EVs owing to the amphipathic property (Dominkus et al., 2018; Simonsen, 2019). Yet the extent of self‐aggregation is generally unknown, and further investigation is highly needed (Russell et al., 2019). Reagent controls with LMD concentrations of 1 μM, 2 μM, 4 μM, and 8 μM were analyzed on the nFCM for all the four LMDs, and the bivariate dot‐plots of LMD fluorescence versus SSC are displayed in Figure 4a‐d. It can be seen that self‐aggregation occurred to all the four LMDs tested, and PKH67 and PKH26 formed four to eight‐fold more aggregates than di‐8‐ANEPPS and DiI. Representative TEM images for 8 μM LMD confirmed the presence of self‐aggregates (Figure 4e). For an easy comparison, the event number detected in 1 min for each LMD sample and the normalized median FL intensity of fluorescent events are plotted in Figure 4f,g, respectively. Whereas the event rate increased with the LMD concentration, the particle fluorescence intensity remained constant for all the four LMDs. These data implied that the particle size of self‐aggregated micelles was in a defined size range, and further increase in LMD concentration would only lead to increased number of aggregates rather than the increased particle size.

Comparing Figures 4 with 3, we can see that the self‐aggregated micelles of LMD exhibited comparable SSC and fluorescence intensity with those of labelled EVs, and are thus hardly be distinguished from each other. Nevertheless, we noticed 8 and 6‐fold more fluorescent particles in 4 μM PKH67 and PKH26 stained EV samples, and 28 and 44‐fold more fluorescent particles in 8 μM di‐8‐ANEPPS and DiI stained EV samples compared to their corresponding reagent controls. It is worth noting that the free dye concentration in the EV sample is actually much lower than that in the reagent control as some LMDs are inserted into the EV membrane. Taking together, the effect of LMD self‐aggregation can be negligible at an optimal staining condition.

### 3.5 LMD labelling of EVs isolated from platelet‐free‐plasma

**FIGURE 5:**
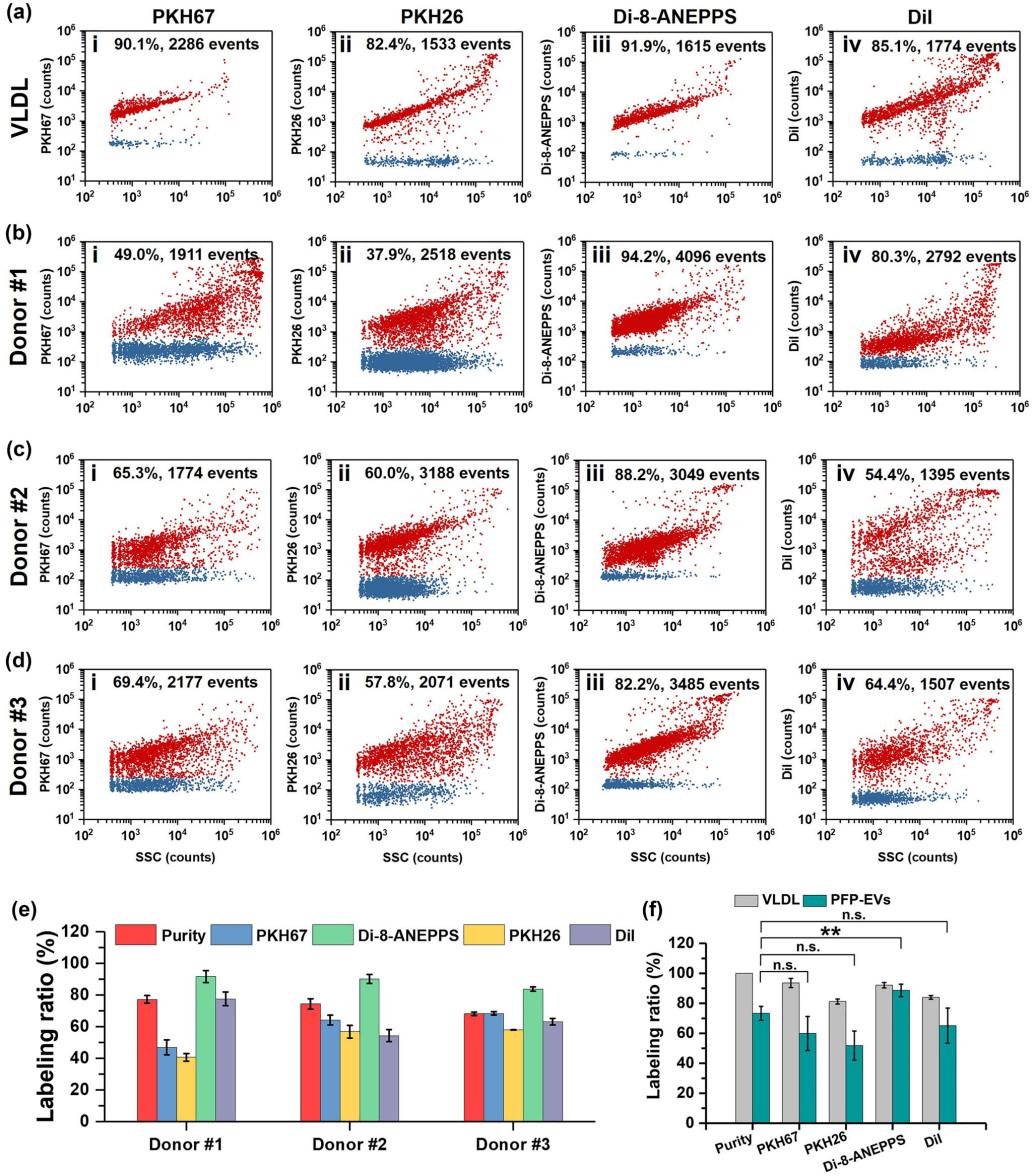
labelling ratio of LMDs for VLDL and EV preparations from PFP. (a) Bivariate dot‐plots of LMD fluorescence versus SSC for VLDL labelled by PKH67 (i), PKH26 (ii), di‐8‐ANEPPS (iii) and DiI (iv). (b‐d) Bivariate dot‐plots of LMD fluorescence versus SSC for EV preparations from PFP of three healthy donors upon labelling with 4 μM PKH67 (i), 4 μM PKH26 (ii), 8 μM di‐8‐ANEPPS (iii), and 8 μM DiI (iv). (e) Purity and labelling ratio of PFP‐EVs of every donor labelled by four LMDs (*n* = 3, mean ± s.d.). (f) Comparison of LMD labelling efficiency between VLDL and PFP‐EVs. Group differences between EV purity and the average labelling ratio of three donors by different LMDs were tested by one‐way ANOVA analysis followed by post‐hoc Bonferroni's test for multiple comparison. *p*‐Value of <0.05 was considered statistically significant. *p*<0.05, *; *p*<0.01, **; *p*<0.001, ***.

Non‐vesicular contaminants including protein aggregates and lipoproteins are often co‐isolated with plasma‐derived EVs. In the present study, VLDL that overlaps significantly with EVs in particle size and density was used as the model system to study the LMD labelling efficiency to lipoproteins. The bivariate dot‐plots of LMD fluorescence versus SSC for VLDL stained with 4 μM PKH67, 4 μM PKH26, 8 μM di‐8‐ANEPPS, and 8 μM DiI are shown in Figure 5a, with labelling ratios of 90.1%, 82.4%, 91.9%, and 85.1%, respectively. The bivariate dot‐plots of LMD fluorescence versus SSC for unlabelled VLDL are shown in Figure S8a. Obviously, VLDL can be almost 100% labelled by all the LMDs. These results verified that when LMDs are applied to label EVs isolated from plasma, attention needs to be paid to the existence of lipoprotein contaminants such as VLDL.

Then EVs isolated upon ultracentrifugation from PFP of three healthy donors were labelled with four LMDs. The bivariate dot‐plots of LMD fluorescence versus SSC are displayed in Figure 5b‐d, and the data for unlabelled PFP‐EVs control are shown in Figure S8b. For an easy comparison, the labelling ratios of different LMDs for different donors are plotted in Figure 5e along with the purity measured by Triton X‐100 lysis assay. Three replicates were conducted for each experiment. It should be kept in mind that defect remains for using Triton X‐100 treatment to assess purity of PFP‐EV preparations as both EVs and lipoproteins could be disrupted after Triton X‐100 treatment (Botha et al., 2022; Tian et al., 2020), the tolerance of each type of lipoproteins to Triton X‐100 treatment is currently unknown, and the population ratio of each type of lipoproteins in a PFP‐EV preparation is also unknown. On the other hand, the lack of a universal EV protein marker for EVs (Thery et al., 2018; Tian et al., 2022, 2018) and the very low ratio of ApoB‐positive VLDL particles (14.4 ± 3.6%) (Figure S9) implicate that antibody staining of EVs (e.g., tetraspanins) and/or lipoproteins (e.g., ApoB) cannot distinguish between EVs and lipoproteins. As density gradient ultracentrifugation can separate EVs and non‐vesicular particles (Jeppesen et al., 2019), density gradient ultracentrifugation was carried out to assess the purity of PFP‐EVs. The results shown in Figure S10 indicate that the particle concentrations of low‐density fractions (fraction 1–6) accounted for 75.1% in total quantity, which was comparable with the purity measured by Triton X‐100 treatment (Donor #3, 68.2% ± 1.1, *n* = 3). Therefore, in the absence of a perfect approach, Triton X‐100 treatment could serve as a simple and effective strategy for the purity assessment of PFP‐EVs. Figure 5e shows that the labelling efficiency of PFP‐EVs varied with the LMDs and donors owing to the individual difference in the composition of plasma. Remarkably, the labelling ratio of di‐8‐ANEPPS were higher than 80% for all four LMDs which were even higher than the purity of EVs. These results implicate that besides EV labelling, di‐8‐ANEPPS also efficiently labelled lipoprotein contaminants. The purity and LMD labelling ratio of VLDL and PFP‐EVs (combined results of three healthy donors) are plotted together for an easy comparison (Figure 5f). Clearly, higher labelling ratios were achieved for VLDL in comparison with PFP‐EVs for PKH67, PKH26, and DiI, whereas comparable preference to VLDL and PFP‐EVs was observed for di‐8‐ANEPPS. In view of the high labelling ratio for lipoproteins, it is worth noting that labelled particles may not necessarily be EVs owing to the existence of lipoproteins.

### 3.6 LMP labelling of EVs isolated from cultured cells and plasma

**FIGURE 6:**
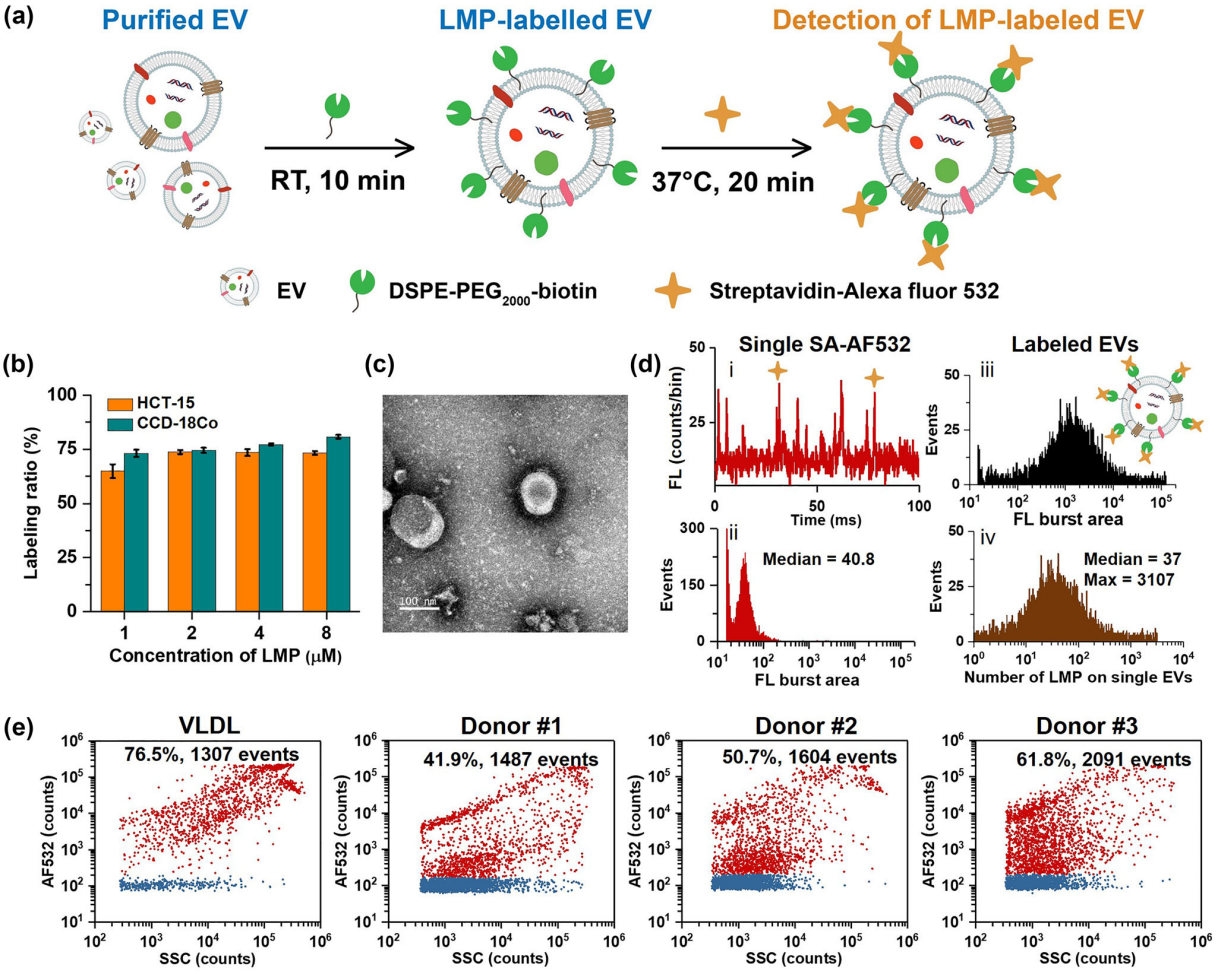
Characterization of LMP labelling for EVs isolated from cultured cells and PFP. (a) Scheme of EV labelling using DSPE‐PEG_2000‐_biotin and streptavidin‐Alexa Fluor 532 and characterization by nFCM. (b) Bar graph of LMP labelling ratios for EVs isolated from CCCM of HCT‐15 and CCD‐18Co cell lines at different LMP concentrations. (c) Representative TEM micrograph of HCT‐15 EVs labelled with 8 μM DSPE‐PEG_2000_‐biotin. The scale bar is 100 nm. (d) Representative fluorescence burst trace (i) and fluorescence distribution histogram (ii) for streptavidin‐AF 532 molecules; fluorescence histograms of HCT‐15 EVs labelled with 8 μM DSPE‐PEG_2000_‐biotin and streptavidin‐AF 532 (iii) and the distribution histogram of the number of LMPs inserted on single EVs. (e) Bivariate dot‐plots of AF532 fluorescence versus SSC for VLDL and PFP‐EVs isolated from three healthy donors upon DSPE‐PEG_2000_‐biotin and streptavidin‐Alexa Fluor 532 labelling.

Numerous LMPs have been designed and synthesized for membrane‐based EV capture and purification (Liu et al., 2022; Sun et al., 2022; Wan et al., 2017). However, the efficiency and selectivity of EV labelling by LMP has not been systematically evaluated. In present study, DSPE‐PEG_2000_‐biotin was synthesized and served as a model LMP. DSPE is able to insert into membrane structures spontaneously, while the PEG spacer for improved reagent solubility and biotin tag for subsequent capture by NeutraAvidin‐coated magnetic beads (Wan et al., 2017). Here, the LMP‐labelled EVs were fluorescently stained with streptavidin‐Alexa Fluor 532 (AF532) and analyzed on the nFCM (Figure 6a). EVs isolated from HCT‐15 and CCD‐18Co cell lines were tested and the bivariate results of AF532 fluorescence versus SSC are shown in Figure S11a,b. The bar graph shown in Figure 6b indicates that the labelling ratios obtained at four different DSPE‐PEG_2000_‐biotin concentrations were almost constant and comparable to the corresponding purities of EVs. Figure S11c,d suggests that compared to PKH dyes, DSPE‐PEG_2000_‐biotin is less likely to form self‐aggregated micelles, and the aggregation performance was comparable to di‐8‐ANEPPS and DiI. TEM image shown in Figure 6c indicates that HCT‐15 EVs could maintain their integrity upon LMP labelling.

Taking advantage of the excellent sensitivity of nFCM in detecting single streptavidin‐AF532 molecules, we measured the number of LMPs inserted into EV membrane. Figure 6d (i, ii) shows the representative fluorescence burst traces and fluorescence distribution histogram of individual streptavidin‐AF532 molecules acquired on the nFCM. By dividing the fluorescence intensity of single EVs upon DSPE‐PEG_2000_‐biotin and streptavidin‐AF532 labelling by the median fluorescence intensity of single streptavidin‐AF532 molecules, the copy number of LMPs inserted on EV surface can be obtained. We can see from the distribution shown in Figure 6d (iv) that there existed a large variation in the number of LMPs inserted on single EVs, which varied from 0 to 3107 with the median value of 37. This large heterogeneity may be ascribed to the diversity of surface area and lipid composition of EVs. It should be noted that a single streptavidin molecule can bind up to 4 biotin molecules. Nevertheless, the measured insertion number provides instructive information for membrane labelling by LMD/LMP.

The labelling conditions of DSPE‐PEG_2000_‐biotin towards VLDL and PFP‐EVs of three donors are illustrated with the bivariate dot‐plots of AF532 fluorescence versus SSC as shown in Figure 6e. We can see that the labelling ratios were 76.5%, 41.9%, 50.7%, and 61.8% for VLDL and PFP‐EVs of donor #1‐3, respectively. Comparing to the corresponding purity of donor EVs shown in Figure 5e, it is clear that neither the VLDL nor the PFP‐EVs were completely labelled by DSPE‐PEG_2000_‐biotin. Thus, the labelling efficiency of EVs by LMP differed between EVs isolated from cultured cells and plasma, and also varied with individuals. Hence cautions should be taken when conclusions are drawn from the downstream molecular analysis of isolated EVs from human plasma.

### 3.7 Potential influence of lipid membrane labelling on surface protein immunophenotyping of EVs

**FIGURE 7:**
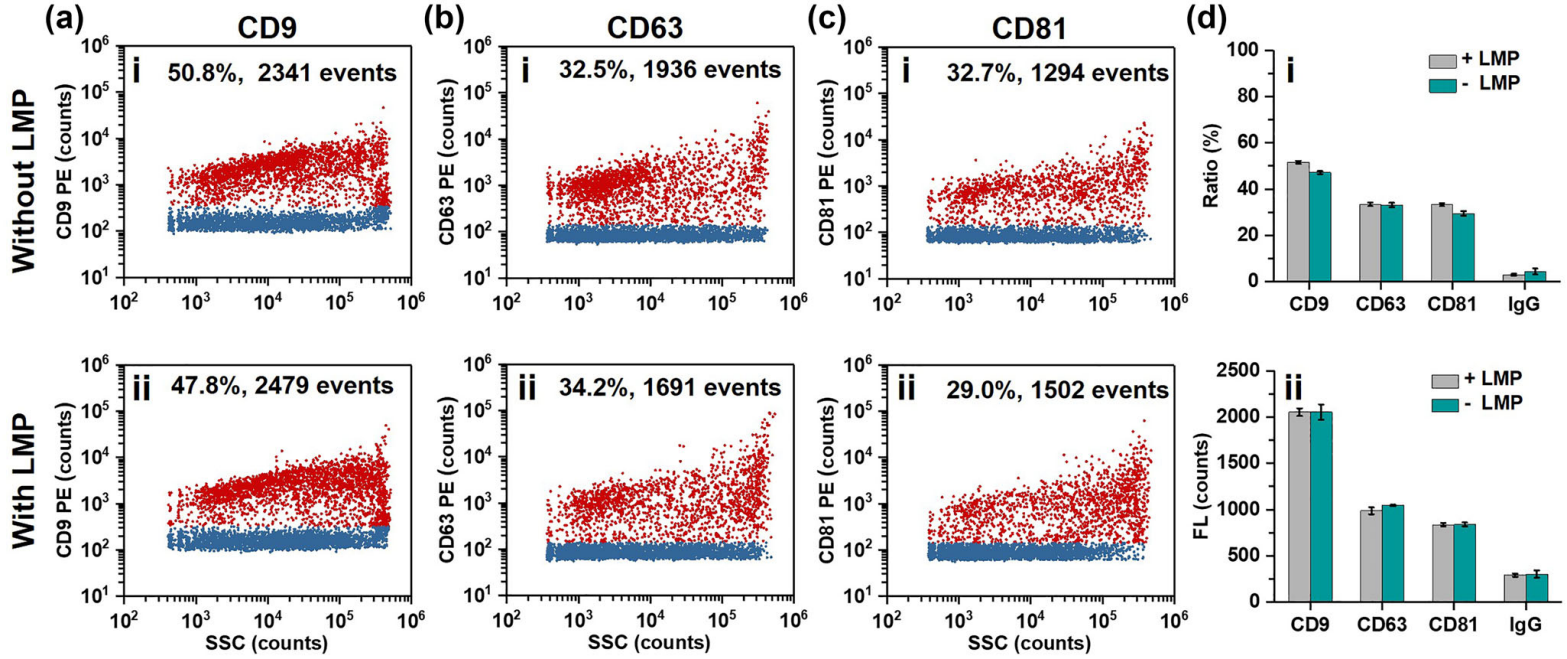
Surface‐protein profiling of EVs isolated from the CCCM of HCT‐15 cell line by ultracentrifugation with or without LMP. (a‐c) Bivariate dot‐plots of immunofluorescence versus SSC for EVs stained with antibodies against CD9 (a), CD63 (b), and CD81 (c) with (i) or without (ii) DSPE‐PEG_2000_‐biotin labelling. (d) Labelling ratio (i) and median immunofluorescence of single EV (ii) with or without DSPE‐PEG_2000_‐biotin labelling.

In EV‐flow cytometry, lipid membrane labelling is often used for fluorescence triggering to enable the discrimination of more EVs against background. So that surface protein markers can then be measured to identify phenotypic subsets of EVs (Panagopoulou et al., 2020; Russell et al., 2019; van der Vlist et al., 2012). To explore whether EV membrane labelling exert steric hindrance effect on surface protein analysis or could simply impair the functionality of EVs, the quantity of classical EV surface proteins CD9, CD63, and CD81 was measured with PE‐conjugated antibodies after labelling with DSPE‐PEG_2000_‐biotin. Figure 7a‐c shows the bivariate dot‐plots of PE‐conjugated antibody fluorescence versus SSC for EVs isolated from the CCCM of HCT‐15 cells without or with LMP insertion. The bivariate dot‐plots for isotype control are shown in Figure S12. The bar graphs shown in Figure 7d indicate that neither the protein labelling ratio nor the median immunofluorescence changed significantly with DSPE‐PEG_2000_‐biotin insertion. These data proved that LMP or LMD insertion into EV membrane is compatible with fluorescent immunostaining.

## 4 DISCUSSION

Despite the indispensability of lipid membrane labelling in the exploration of the physiological function and isolation of EVs, the labelling efficiency of LMD/LMP and the interference of self‐aggregation and lipoproteins have not been systematically characterized. Here, we established a high‐throughput method to evaluate the labelling performance of LMD/LMP on the single‐particle basis by nFCM. In contrast to the isolation efficiency measured by ensemble techniques such as the mass fraction of RNA extracted from the captured EVs over total EVs (Wan et al., 2017), the labelling ratio obtained by detecting LMD/LMP‐positive EVs out of the total EV population represents a more straightforward and quantitative measure. We first demonstrated that PKH67, PKH26, di‐8‐ANEPPS, and DiI exhibit a favourable (nearly 100%) labelling for synthetic liposomes, which usually have simple lipid composition and less contamination of impurity particles. Lipidomics study revealed that the lipid membrane of EV preparations from the conditioned cell culture medium of HCT‐15 cells by ultracentrifugation consisted of 23 lipid classes (PC, PE, PS, and SM were the most abundant four classes) and more than 700 lipid species. With respect to the ∼90% purity of EVs isolated from HCT‐15 cells, it was identified that PKH67 and PKH26 could only label ∼60%–70% of EVs even at high LMD concentrations, and excessive PKH26 could cause damage to the EV structure. However, it is interesting to note that 8 μM PKH67 could label almost all the EVs isolated from CCD‐18Co cells on the contrary, which implicating their different lipid compositions from EVs secreted by HCT‐15 cells. In contrast to PKH dyes, 1 to 8 μM of di‐8‐ANEPPS and 8 μM DiI achieved efficient and uniform labelling of EVs isolated from both the HCT‐15 and CCD‐18Co cells. The different labelling performance on the same EV samples exhibited by different LMDs suggested the molecular structures of LMDs have important impact on membrane labelling (Faller, 2016; Leung et al., 2017; Munter et al., 2018). The excellent labelling performances of di‐8‐ANEPPS has been reported by several recent studies, which may be attributed to the net negative charge of the head group and the insensitivity to lipid compositions and membrane dipole (Andronico et al., 2021; de Rond et al., 2018; Stoner et al., 2016).

Both the nFCM and TEM examination of the EV‐free reagent control samples confirmed that all the four LMDs can self‐aggregate and form micelles that fluoresce and hard to differentiate from labelled EVs. As the LMD concentration went up, the number of self‐aggregation micelles increased while the particles size and fluorescence intensity remained constant. PKH67 and PKH26 tend to form more aggregated micelles than di‐8‐ANEPPS and DiI. Our observation agreed with the earlier report that lipid molecules possessing longer alkyl chains are easier to form self‐aggregated micelles (Simonsen, 2019). Nevertheless, the aggregated micelles only took a small fraction of all the fluorescent particles upon di‐8‐ANEPPS and DiI labelling once the free LMDs were washed away by ultracentrifugation.

The presence of lipoproteins is another source of error when labelling EVs with LMDs, especially for plasma EVs. Here, we present solid evidence that LMDs can label almost all the VLDL particles and the labelled VLDL exhibit comparable FL and SSC signals with labelled EVs. Since there has been no specific marker identified for EVs or lipoproteins, it is currently impracticable to completely distinguish lipoproteins from EVs based on flow cytometry analysis (Botha et al., 2022). Thus combined utilization of different separation strategies, such as UC followed by SEC or gradient density UC is recommended to remove the contaminants to obtain more pure plasma EV sample (Jeppesen et al., 2019; Takov et al., 2019; Zhang et al., 2018). Furthermore, the number of LMPs inserted into the membrane of single EVs was measured for the first time, which provides instructive information of EV membrane labelling. Moreover, we demonstrated that surface insertion of lipid probes had no impact on immunophenotyping of EVs.

In conclusion, by analyzing single particles with side scatter triggering and concurrent fluorescence detection, nFCM revealed that none of tested PKH67, PKH26, di‐8‐ANEPPS, DiI, and DSPE‐PEG_2000_‐biotin bind to all EVs and only EVs. Among which, di‐8‐ANEPPS performs the best in terms of bright and uniform labelling as well as less degree of self‐aggregation. However, its broad emission spectrum may pose a challenge when designing multicolour panels. In summary, the labelling efficiency of any newly synthesized LMD/LMP needs to be evaluated to enable effective detection or isolation of EVs. Careful control should be carried out for lipophilic membrane dye staining experiments, and conclusions based on LMD fluorescence or LMP capture should be interpreted with caution.

## AUTHOR CONTRIBUTIONS

**Chen Chen**: Conceptualization; Data curation; Formal analysis; Methodology; Writing‐original draft; Writing‐review & editing. **Niangui Cai**: Formal analysis; Investigation; Methodology; Writing‐review & editing. **Qian Niu**: Data curation; Formal analysis; Investigation. **Ye Tian**: Conceptualization; Data curation; Methodology. **Yunyun Hu**: Formal analysis; Methodology. **Xiaomei Yan**: Conceptualization; Formal analysis; Funding acquisition; Methodology; Project administration; Resources; Supervision; Writing‐original draft; Writing‐review & editing.

## CONFLICT OF INTEREST STATEMENT

X.Y. declares competing financial interests as a cofounder of NanoFCM Inc., a company committed to commercializing the nano‐flow cytometry (nFCM) technology.

## Supporting information

Supplementary Information

## References

[jev212351-bib-0001] Andronico, L. A. , Jiang, Y. F. , Jung, S. R. , Fujimoto, B. S. , Vojtech, L. , & Chiu, D. T. (2021). Sizing extracellular vesicles using membrane dyes and a single molecule‐sensitive flow analyzer. Analytical Chemistry, 93, 5897–5905. 33784071 10.1021/acs.analchem.1c00253 PMC10243643

[jev212351-bib-0002] Botha, J. , Handberg, A. , & Simonsen, J. B. (2022). Lipid‐based strategies used to identify extracellular vesicles in flow cytometry can be confounded by lipoproteins: Evaluations of annexin V, lactadherin, and detergent lysis. Journal of Extracellular Vesicles, 11, e12200. 35362259 10.1002/jev2.12200 PMC8971177

[jev212351-bib-0003] Chen, C. X. , Zhu, S. B. , Wang, S. , Zhang, W. Q. , Cheng, Y. , & Yan, X. M. (2017). Multiparameter quantification of liposomal nanomedicines at the single‐particle level by high‐sensitivity flow cytometry. ACS Applied Materials & Interfaces, 9, 13913–13919. 28374584 10.1021/acsami.7b01867

[jev212351-bib-0004] Chen, F. , Chen, J. , Yang, L. , Liu, J. , Zhang, X. , Zhang, Y. , Tu, Q. , Yin, D. , Lin, D. , Wong, P. P. , Huang, D. , Xing, Y. , Zhao, J. , Li, M. , Liu, Q. , Su, F. , Su, S. , & Song, E. (2019). Extracellular vesicle‐packaged HIF‐1alpha‐stabilizing lncRNA from tumour‐associated macrophages regulates aerobic glycolysis of breast cancer cells. Nature Cell Biology, 21, 498–510. 30936474 10.1038/s41556-019-0299-0

[jev212351-bib-0005] Choi, D. , Montermini, L. , Jeong, H. , Sharma, S. , Meehan, B. , & Rak, J. (2019). Mapping subpopulations of cancer cell‐derived extracellular vesicles and particles by nano‐flow cytometry. ACS Nano, 13, 10499–10511. 31469961 10.1021/acsnano.9b04480

[jev212351-bib-0006] de Rond, L. , van der Pol, E. , Hau, C. M. , Varga, Z. , Sturk, A. , van Leeuwen, T. G. , Nieuwland, R. , & Coumans, F. A. W. (2018). Comparison of generic fluorescent markers for detection of extracellular vesicles by flow cytometry. Clinical Chemistry, 64, 680–689. 29453194 10.1373/clinchem.2017.278978

[jev212351-bib-0007] Dehghani, M. , Gulvin, S. M. , Flax, J. , & Gaborski, T. R. (2020). Systematic evaluation of PKH labelling on extracellular vesicle size by nanoparticle tracking analysis. Scientific Reports, 10, 9533. 32533028 10.1038/s41598-020-66434-7 PMC7293335

[jev212351-bib-0008] Di, H. X. , Zeng, E. Z. , Zhang, P. J. , Liu, X. H. , Zhang, C. , Yang, J. , & Liu, D. B. (2019). General approach to engineering extracellular vesicles for biomedical analysis. Analytical Chemistry, 91, 12752–12759. 31529961 10.1021/acs.analchem.9b02268

[jev212351-bib-0009] Ding, D. , Li, K. , Liu, B. , & Tang, B. Z. (2013). Bioprobes based on AIE fluorogens. Accounts of Chemical Research, 46, 2441–2453. 23742638 10.1021/ar3003464

[jev212351-bib-0010] Dominkus, P. P. , Stenovec, M. , Sitar, S. , Lasic, E. , Zorec, R. , Plemenitas, A. , Zagar, E. , Kreft, M. , & Lenassi, M. (2018). PKH26 labeling of extracellular vesicles: Characterization and cellular internalization of contaminating PKH26 nanoparticles. BBA‐Biomembranes, 1860, 1350–1361. 29551275 10.1016/j.bbamem.2018.03.013

[jev212351-bib-0011] Faller, R. (2016). Molecular modeling of lipid probes and their influence on the membrane. BBA‐Biomembranes, 1858, 2353–2361. 26891817 10.1016/j.bbamem.2016.02.014

[jev212351-bib-0012] Gardiner, C. , Shaw, M. , Hole, P. , Smith, J. , Tannetta, D. , Redman, C. W. , & Sargent, I. L. (2014). Measurement of refractive index by nanoparticle tracking analysis reveals heterogeneity in extracellular vesicles. Journal of Extracell Vesicles, 3, 25361. 10.3402/jev.v3.25361 PMC4247498 25425324

[jev212351-bib-0013] Grange, C. , Tapparo, M. , Bruno, S. , Chatterjee, D. , Quesenberry, P. J. , Tetta, C. , & Camussi, G. (2014). Biodistribution of mesenchymal stem cell‐derived extracellular vesicles in a model of acute kidney injury monitored by optical imaging. International Journal of Molecular Medicine, 33, 1055–1063. 24573178 10.3892/ijmm.2014.1663 PMC4020482

[jev212351-bib-0014] Herrmann, I. K. , Wood, M. J. A. , & Fuhrmann, G. (2021). Extracellular vesicles as a next‐generation drug delivery platform. Nature Nanotechnology, 16, 748–759. 10.1038/s41565-021-00931-2 34211166

[jev212351-bib-0015] Hussey, G. S. , Pineda Molina, C. , Cramer, M. C. , Tyurina, Y. Y. , Tyurin, V. A. , Lee, Y. C. , El‐Mossier, S. O. , Murdock, M. H. , Timashev, P. S. , Kagan, V. E. , & Badylak, S. F. (2020). Lipidomics and RNA sequencing reveal a novel subpopulation of nanovesicle within extracellular matrix biomaterials. Science Advances, 6, eaay4361. 32219161 10.1126/sciadv.aay4361 PMC7083606

[jev212351-bib-0016] Jeppesen, D. K. , Fenix, A. M. , Franklin, J. L. , Higginbotham, J. N. , Zhang, Q. , Zimmerman, L. J. , Liebler, D. C. , Ping, J. , Liu, Q. , Evans, R. , Fissell, W. H. , Patton, J. G. , Rome, L. H. , Burnette, D. T. , & Coffey, R. J. (2019). Reassessment of exosome composition. Cell, 177, 428–445. 30951670 10.1016/j.cell.2019.02.029 PMC6664447

[jev212351-bib-0017] Kalluri, R. , & LeBleu, V. S. (2020). The biology, function, and biomedical applications of exosomes. Science, 367, eaau6977. 32029601 10.1126/science.aau6977 PMC7717626

[jev212351-bib-0018] Kamei, N. , Nishimura, H. , Matsumoto, A. , Asano, R. , Muranaka, K. , Fujita, M. , Takeda, M. , Hashimoto, H. , & Takeda‐Morishita, M. (2021). Comparative study of commercial protocols for high recovery of high‐purity mesenchymal stem cell‐derived extracellular vesicle isolation and their efficient labeling with fluorescent dyes. Nanomedicine‐Nanotechnology Biology and Medicine, 35, 102396. 33864911 10.1016/j.nano.2021.102396

[jev212351-bib-0019] Kamerkar, S. , LeBleu, V. S. , Sugimoto, H. , Yang, S. , Ruivo, C. F. , Melo, S. A. , Lee, J. J. , & Kalluri, R. (2017). Exosomes facilitate therapeutic targeting of oncogenic KRAS in pancreatic cancer. Nature, 546, 498–503. 28607485 10.1038/nature22341 PMC5538883

[jev212351-bib-0020] Karimi, N. , Cvjetkovic, A. , Jang, S. C. , Crescitelli, R. , Feizi, M. A. H. , Nieuwland, R. , Lotvall, J. , & Lasser, C. (2018). Detailed analysis of the plasma extracellular vesicle proteome after separation from lipoproteins. Cellular and Molecular Life Sciences, 75, 2873–2886. 29441425 10.1007/s00018-018-2773-4 PMC6021463

[jev212351-bib-0021] Lai, C. P. , Kim, E. Y. , Badr, C. E. , Weissleder, R. , Mempel, T. R. , Tannous, B. A. , & Breakefield, X. O. (2015). Visualization and tracking of tumour extracellular vesicle delivery and RNA translation using multiplexed reporters. Nature Communications, 6, 8029. 10.1038/ncomms8029 PMC4435621 25967391

[jev212351-bib-0022] Leung, S. S. W. , & Thewalt, J. (2017). Link between fluorescent probe partitioning and molecular order of liquid ordered‐liquid disordered membranes. Journal of Physical Chemistry B, 121, 1176–1185. 28145724 10.1021/acs.jpcb.6b09325

[jev212351-bib-0023] Lian, H. , He, S. , Chen, C. , & Yan, X. (2019). Flow cytometric analysis of nanoscale biological particles and organelles. Annual Review of Analytical Chemistry, 12, 389–409. 10.1146/annurev-anchem-061318-115042 30978294

[jev212351-bib-0024] Lide, D. R. (2010). CRC handbook of chemistry and physics, 12–153, 90th ed.; CRC Press.

[jev212351-bib-0025] Liu, X. H. , Zong, Z. Y. , Liu, X. Z. , Li, Q. , Li, A. , Xu, C. , & Liu, D. B. (2022). Stimuli‐mediated specific isolation of exosomes from blood plasma for high‐throughput profiling of cancer biomarkers. Small Methods, 6, 2101234. 10.1002/smtd.202101234 35174989

[jev212351-bib-0026] Mathieu, M. , Martin‐Jaular, L. , Lavieu, G. , & Thery, C. (2019). Specificities of secretion and uptake of exosomes and other extracellular vesicles for cell‐to‐cell communication. Nature Cell Biology, 21, 9–17. 30602770 10.1038/s41556-018-0250-9

[jev212351-bib-0027] Melo, S. A. , Luecke, L. B. , Kahlert, C. , Fernandez, A. F. , Gammon, S. T. , Kaye, J. , LeBleu, V. S. , Mittendorf, E. A. , Weitz, J. , Rahbari, N. , Reissfelder, C. , Pilarsky, C. , Fraga, M. F. , Piwnica‐Worms, D. , & Kalluri, R. (2015). Glypican‐1 identifies cancer exosomes and detects early pancreatic cancer. Nature, 523, 177–182. 26106858 10.1038/nature14581 PMC4825698

[jev212351-bib-0028] Midekessa, G. , Godakumara, K. , Dissanayake, K. , Hasan, M. M. , Reshi, Q. U. , Rinken, T. , & Fazeli, A. (2021). Characterization of extracellular vesicles labelled with a lipophilic dye using fluorescence nanoparticle tracking analysis. Membranes, 11, 779. 34677545 10.3390/membranes11100779 PMC8539200

[jev212351-bib-0029] Morales‐Kastresana, A. , Telford, B. , Musich, T. A. , McKinnon, K. , Clayborne, C. , Braig, Z. , Rosner, A. , Demberg, T. , Watson, D. C. , Karpova, T. S. , Freeman, G. J. , DeKruyff, R. H. , Pavlakis, G. N. , Terabe, M. , Robert‐Guroff, M. , Berzofsky, J. A. , & Jones, J. C. (2017). Labeling extracellular vesicles for nanoscale flow cytometry. Scientific Reports, 7, 1878. 28500324 10.1038/s41598-017-01731-2 PMC5431945

[jev212351-bib-0030] Munter, R. , Kristensen, K. , Pedersbaek, D. , Larsen, J. B. , Simonsen, J. B. , & Andresen, T. L. (2018). Dissociation of fluorescently labeled lipids from liposomes in biological environments challenges the interpretation of uptake studies. Nanoscale, 10, 22720–22724. 30488936 10.1039/c8nr07755j

[jev212351-bib-0031] Oh, C. K. , Dolatabadi, N. , Cieplak, P. , Diaz‐Meco, M. T. , Moscat, J. , Nolan, J. P. , Nakamura, T. , & Lipton, S. A. (2022). S‐nitrosylation of p62 inhibits autophagic flux to promote alpha‐synuclein secretion and spread in Parkinson's disease and Lewy body dementia. The Journal of Neuroscience, 42, 3011–3024. 35169022 10.1523/JNEUROSCI.1508-21.2022 PMC8985870

[jev212351-bib-0032] Osella, S. , & Knippenberg, S. (2021). The influence of lipid membranes on fluorescent probes' optical properties. BBA‐Biomembranes, 1863, 183494. 33129783 10.1016/j.bbamem.2020.183494

[jev212351-bib-0033] Panagopoulou, M. S. , Wark, A. W. , Birch, D. J. S. , & Gregory, C. D. (2020). Phenotypic analysis of extracellular vesicles: A review on the applications of fluorescence. Journal of Extracellular Vesicles, 9, 1710020. 32002172 10.1080/20013078.2019.1710020 PMC6968689

[jev212351-bib-0034] Park, D. J. , Duggan, E. , Ho, K. , Dorschner, R. A. , Dobke, M. , Nolan, J. P. , & Eliceiri, B. P. (2022). Serpin‐loaded extracellular vesicles promote tissue repair in a mouse model of impaired wound healing. Journal of Nanobiotechnology, 20, 474. 36335351 10.1186/s12951-022-01656-7 PMC9636779

[jev212351-bib-0035] Russell, A. E. , Sneider, A. , Witwer, K. W. , Bergese, P. , Bhattacharyya, S. N. , Cocks, A. , Cocucci, E. , Erdbrugger, U. , Falcon‐Perez, J. M. , Freeman, D. W. , Gallagher, T. M. , Hu, S. S. , Huang, Y. Y. , Jay, S. M. , Kano, S. , Lavieu, G. , Leszczynska, A. , Llorente, A. M. , Lu, Q. , … Vader, P. (2019). Biological membranes in EV biogenesis, stability, uptake, and cargo transfer: an ISEV position paper arising from the ISEV membranes and EVs workshop. Journal of Extracellular Vesicles, 8, 1684862. 31762963 10.1080/20013078.2019.1684862 PMC6853251

[jev212351-bib-0036] Sandau, U. S. , Duggan, E. , Shi, X. , Smith, S. J. , Huckans, M. , Schutzer, W. E. , Loftis, J. M. , Janowsky, A. , Nolan, J. P. , & Saugstad, J. A. (2020). Methamphetamine use alters human plasma extracellular vesicles and their microRNA cargo: An exploratory study. Journal of Extracell Vesicles, 10, e12028. 10.1002/jev2.12028 PMC7890470 33613872

[jev212351-bib-0037] Shpigelman, J. , Lao, F. S. , Yao, S. , Li, C. , Saito, T. , Sato‐Kaneko, F. , Nolan, J. P. , Shukla, N. M. , Pu, M. , Messer, K. , Cottam, H. B. , Carson, D. A. , Corr, M. , & Hayashi, T. (2021). Generation and application of a reporter cell line for the quantitative screen of extracellular vesicle release. Frontiers in Pharmacology, 12, 668609. 33935791 10.3389/fphar.2021.668609 PMC8085554

[jev212351-bib-0038] Simonsen, J. B. (2017). What are we looking at? Extracellular vesicles, lipoproteins, or both? Circulation Research, 121, 920–922. 28963190 10.1161/CIRCRESAHA.117.311767

[jev212351-bib-0039] Simonsen, J. B. (2019). Pitfalls associated with lipophilic fluorophore staining of extracellular vesicles for uptake studies. Journal of Extracellular Vesicles, 8, 1582237. 30815239 10.1080/20013078.2019.1582237 PMC6383605

[jev212351-bib-0040] Skotland, T. , Sagini, K. , Sandvig, K. , & Llorente, A. (2020). An emerging focus on lipids in extracellular vesicles. Advanced Drug Delivery Reviews, 159, 308–321. 32151658 10.1016/j.addr.2020.03.002

[jev212351-bib-0041] Stoner, S. A. , Duggan, E. , Condello, D. , Guerrero, A. , Turk, J. R. , Narayanan, P. K. , & Nolan, J. P. (2016). High sensitivity flow cytometry of membrane vesicles. Cytometry Part A, 89a, 196–206. 10.1002/cyto.a.22787 26484737

[jev212351-bib-0042] Sun, N. , Tran, B. V. , Peng, Z. S. , Wang, J. , Zhang, C. , Yang, P. , Zhang, T. X. , Widjaja, J. , Zhang, R. Y. , Xia, W. X. , Keir, A. , She, J. W. , Yu, H. H. , Shyue, J. J. , Zhu, H. G. , Agopian, V. G. , Pei, R. J. , Tomlinson, J. S. , Toretsky, J. A. , … Zhu, Y. (2022). Coupling lipid labeling and click chemistry enables isolation of extracellular vesicles for noninvasive detection of oncogenic gene alterations. Advanced Science, 9, 2105853. 35486030 10.1002/advs.202105853 PMC9108594

[jev212351-bib-0043] Takov, K. , Yellon, D. M. , & Davidson, S. M. (2017). Confounding factors in vesicle uptake studies using fluorescent lipophilic membrane dyes. Journal of Extracellular Vesicles, 6, 1388731. 29184625 10.1080/20013078.2017.1388731 PMC5699187

[jev212351-bib-0044] Takov, K. , Yellon, D. M. , & Davidson, S. M. (2019). Comparison of small extracellular vesicles isolated from plasma by ultracentrifugation or size‐exclusion chromatography: yield, purity and functional potential. Journal of Extracell Vesicles, 8, 1560809. 10.1080/20013078.2018.1560809 PMC6327926 30651940

[jev212351-bib-0045] Thery, C. , Witwer, K. W. , Aikawa, E. , Alcaraz, M. J. , Anderson, J. D. , Andriantsitohaina, R. , Antoniou, A. , Arab, T. , Archer, F. , Atkin‐Smith, G. K. , Ayre, D. C. , Bach, J. M. , Bachurski, D. , Baharvand, H. , Balaj, L. , Baldacchino, S. , Bauer, N. N. , Baxter, A. A. , Bebawy, M. , … Zuba‐Surma, E. K. (2018). Minimal information for studies of extracellular vesicles 2018 (MISEV2018): a position statement of the International Society for Extracellular Vesicles and update of the MISEV2014 guidelines. Journal of Extracellular Vesicles, 7, 1535750. 30637094 10.1080/20013078.2018.1535750 PMC6322352

[jev212351-bib-0046] Tian, Y. , Xue, C. F. , Zhang, W. Q. , Chen, C. X. , Niu, Q. , Ma, L. , Wu, L. A. , & Yan, X. M. (2022). Refractive index determination of individual viruses and small extracellular vesicles in aqueous media using nano‐flow cytometry. Analytical Chemistry, 94, 14299–14307. 36084271 10.1021/acs.analchem.2c02833

[jev212351-bib-0047] Tian, Y. , Gong, M. F. , Hu, Y. Y. , Liu, H. S. , Zhang, W. Q. , Zhang, M. M. , Hu, X. X. , Aubert, D. , Zhu, S. B. , Wu, L. , & Yan, X. M. (2020). Quality and efficiency assessment of six extracellular vesicle isolation methods by nano‐flow cytometry. Journal of Extracellular Vesicles, 9, 1697028. 31839906 10.1080/20013078.2019.1697028 PMC6896440

[jev212351-bib-0048] Tian, Y. , Ma, L. , Gong, M. F. , Su, G. Q. , Zhu, S. B. , Zhang, W. Q. , Wang, S. , Li, Z. B. , Chen, C. X. , Li, L. H. , Wu, L. N. , & Yan, X. M. (2018). Protein profiling and sizing of extracellular vesicles from colorectal cancer patients via flow cytometry. ACS Nano, 12, 671–680. 29300458 10.1021/acsnano.7b07782

[jev212351-bib-0049] van der Pol, E. , Coumans, F. A. W. , Sturk, A. , Nieuwland, R. , & van Leeuwen, T. G. (2014). Refractive index determination of nanoparticles in suspension using nanoparticle tracking analysis. Nano Letters, 14, 6195–6201. 25256919 10.1021/nl503371p

[jev212351-bib-0050] van der Pol, E. , Sturk, A. , van Leeuwen, T. , Nieuwland, R. , Coumans, F. , & Grp, I.‐S.‐V. W. (2018). Standardization of extracellular vesicle measurements by flow cytometry through vesicle diameter approximation. Journal of Thrombosis and Haemostasis, 16, 1236–1245. 29575716 10.1111/jth.14009

[jev212351-bib-0051] van der Pol, E. , de Rond, L. , Coumans, F. A. W. , Gool, E. L. , Boing, A. N. , Sturk, A. , Nieuwland, R. , & van Leeuwen, T. G. (2018). Absolute sizing and label‐free identification of extracellular vesicles by flow cytometry. Nanomedicine‐Nanotechnology Biology and Medicine, 14, 801–810. 29307842 10.1016/j.nano.2017.12.012

[jev212351-bib-0052] van der Pol, E. , Coumans, F. A. W. , Grootemaat, A. E. , Gardiner, C. , Sargent, I. L. , Harrison, P. , Sturk, A. , van Leeuwen, T. G. , & Nieuwland, R. (2014). Particle size distribution of exosomes and microvesicles determined by transmission electron microscopy, flow cytometry, nanoparticle tracking analysis, and resistive pulse sensing. Journal of Thrombosis and Haemostasis, 12, 1182–1192. 24818656 10.1111/jth.12602

[jev212351-bib-0053] van der Vlist, E. J. , Nolte‐’t Hoen, E. N. M. , Stoorvogel, W. , Arkesteijn, G. J. A. , & Wauben, M. H. M. (2012). Fluorescent labeling of nano‐sized vesicles released by cells and subsequent quantitative and qualitative analysis by high‐resolution flow cytometry. Nature Protocols, 7, 1311–1326. 22722367 10.1038/nprot.2012.065

[jev212351-bib-0054] van Niel, G. , D'Angelo, G. , & Raposo, G. (2018). Shedding light on the cell biology of extracellular vesicles. Nature Reviews Molecular Cell Biology, 19, 213–228. 29339798 10.1038/nrm.2017.125

[jev212351-bib-0055] Wan, Y. , Cheng, G. , Liu, X. , Hao, S. J. , Nisic, M. , Zhu, C. D. , Xia, Y. Q. , Li, W. Q. , Wang, Z. G. , Zhang, W. L. , Rice, S. J. , Sebastian, A. , Albert, I. , Belani, C. P. , & Zheng, S. Y. (2017). Rapid magnetic isolation of extracellular vesicles via lipid‐based nanoprobes. Nature Biomedical Engineering, 1, 0058. 10.1038/s41551-017-0058 PMC5618714 28966872

[jev212351-bib-0056] Willms, E. , Cabanas, C. , Mager, I. , Wood, M. J. A. , & Vader, P. (2018). Extracellular vesicle heterogeneity: Subpopulations, isolation techniques, and diverse functions in cancer progression. Frontiers in Immunology, 9, 738. 29760691 10.3389/fimmu.2018.00738 PMC5936763

[jev212351-bib-0057] Xu, R. , Rai, A. , Chen, M. S. , Suwakulsiri, W. , Greening, D. W. , & Simpson, R. J. (2018). Extracellular vesicles in cancer ‐ implications for future improvements in cancer care. Nature Reviews Clinical Oncology, 15, 617–638. 10.1038/s41571-018-0036-9 29795272

[jev212351-bib-0058] Yan, W. , Cao, M. , Ruan, X. , Jiang, L. , Lee, S. , Lemanek, A. , Ghassemian, M. , Pizzo, D. P. , Wan, Y. , Qiao, Y. , Chin, A. R. , Duggan, E. , Wang, D. , Nolan, J. P. , Esko, J. D. , Schenk, S. , & Wang, S. E. (2022). Cancer‐cell‐secreted miR‐122 suppresses O‐GlcNAcylation to promote skeletal muscle proteolysis. Nature Cell Biology, 24, 793–804. 35469018 10.1038/s41556-022-00893-0 PMC9107513

[jev212351-bib-0059] Yang, L. L. , Zhu, S. B. , Hang, W. , Wu, L. , & Yan, X. M. (2009). Development of an ultrasensitive dual‐channel flow cytometer for the individual analysis of nanosized particles and biomolecules. Analytical Chemistry, 81, 2555–2563. 19260698 10.1021/ac802464a

[jev212351-bib-0060] Yong, T. Y. , Zhang, X. Q. , Bie, N. N. , Zhang, H. B. , Zhang, X. T. , Li, F. Y. , Hakeem, A. , Hu, J. , Gan, L. , Santos, H. A. , & Yang, X. L. (2019). Tumor exosome‐based nanoparticles are efficient drug carriers for chemotherapy. Nature Communications, 10, 3838. 10.1038/s41467-019-11718-4 PMC6707218 31444335

[jev212351-bib-0061] Zabeo, D. , Cvjetkovic, A. , Lasser, C. , Schorb, M. , Lotvall, J. , & Hoog, J. L. (2017). Exosomes purified from a single cell type have diverse morphology. Journal of Extracellular Vesicles, 6, 1329476. 28717422 10.1080/20013078.2017.1329476 PMC5505001

[jev212351-bib-0062] Zhang, H. Y. , Freitas, D. , Kim, H. S. , Fabijanic, K. , Li, Z. , Chen, H. Y. , Mark, M. T. , Molina, H. , Martin, A. B. , Bojmar, L. , Fang, J. , Rampersaud, S. , Hoshino, A. , Matei, I. , Kenific, C. M. , Nakajima, M. , Mutvei, A. P. , Sansone, P. , Buehring, W. , … Lyden, D. (2018). Identification of distinct nanoparticles and subsets of extracellular vesicles by asymmetric flow field‐flow fractionation. Nature Cell Biology, 20, 332–343. 29459780 10.1038/s41556-018-0040-4 PMC5931706

[jev212351-bib-0063] Zhu, S. B. , Yang, L. L. , Long, Y. , Gao, M. , Huang, T. X. , Hang, W. , & Yan, X. M. (2010). Size differentiation and absolute quantification of gold nanoparticles via single particle detection with a laboratory‐built high‐sensitivity flow cytometer. Journal of the American Chemical Society, 132, 12176–12178. 20707319 10.1021/ja104052c

[jev212351-bib-0064] Zhu, S. B. , Ma, L. , Wang, S. , Chen, C. X. , Zhang, W. Q. , Yang, L. L. , Hang, W. , Nolan, J. P. , Wu, L. N. , & Yan, X. M. (2014). Light‐scattering detection below the level of single fluorescent molecules for high‐resolution characterization of functional nanoparticles. ACS Nano, 8, 10998–11006. 25300001 10.1021/nn505162u PMC4212780

[jev212351-bib-0065] Zijlstra, A. , & Di Vizio, D. (2018). Size matters in nanoscale communication. Nature Cell Biology, 20, 228–230. 29476154 10.1038/s41556-018-0049-8 PMC6652179

