# Supplementary material for: Quantitative assessment of lipophilic membrane dye‐based labelling of extracellular vesicles by nano‐flow cytometry
Source: J Extracell Vesicles. 2023 Jul 31;12(8):12351. doi: 10.1002/jev2.12351 (PMC10390660; doi:10.1002/jev2.12351)
Supplement: Supplementary file 1 — Supplementary Information [file JEV2-12-12351-s001.docx]

**Supplementary Information**

**Quantitative assessment of lipophilic membrane dye-based labeling of extracellular vesicles by nano-flow cytometry**

Chen Chen, Niangui Cai, Qian Niu, Ye Tian, Yunyun Hu and Xiaomei Yan*

Department of Chemical Biology, MOE Key Laboratory of Spectrochemical Analysis & Instrumentation, Key Laboratory for Chemical Biology of Fujian Province, State Key Laboratory of Physical Chemistry of Solid Surfaces, College of Chemistry and Chemical Engineering, Xiamen University, Xiamen 361005, People's Republic of China

^*^To whom correspondence should be addressed. E-mail: xmyan@xmu.edu.cn

This Supplementary Material contains methods for preparation of PFP, transmission electron microscopy (TEM), Western blotting, and Figures S1-S10.

***Preparation of platelet-free plasma (PFP)***

Peripheral blood was drawn from healthy volunteers who had fasted for at least 12 h at the First Affiliated Hospital of Xiamen University. Informed written consent was obtained from the healthy volunteers and the collection of human blood samples was approved by the Ethics Committee of the First Affiliated Hospital of Xiamen University. Briefly, 2.7 mL blood was collected into BD Vacutainer tubes (363095) containing 0.3 mL of 0.109 M sodium citrate by using 21-gauge needles. After collection, tubes were inverted 4–5 times immediately for proper mixing with anticoagulant. Tubes were transported vertically at room temperature without agitation. And then, the blood samples were centrifuged twice at 2,500 × g for 15 min at room temperature to extract PFP within 2 h of blood collection. PFP was aliquoted and stored at −80°C until further use, and freeze-thawing was avoided as much as possible after that.

***Transmission electron microscopy (TEM)***

A 3-μL aliquot of the EV preparations or self-aggregated particles of LMDs (20 times as much of the dosage for nFCM) was placed on a formvar-/carbon-coated grid and allowed to settle for 2 min. The sample was negative-stained with 2% phosphotungstic acid for 1 min. The grid was imaged with a Tecnai G2 Spirit BioTwin transmission electron microscope operating at 120 kV.

***Western blotting***

The protein concentration of EV preparations was measured using a Pierce ™ BCA protein assay kit (Thermo Fisher, 23227). For each sample, the protein concentration was adjusted to 10 μg/10 μL, and 15 μg of protein was loaded onto a 15% polyacrylamide gel. Following electrophoresis, the proteins were transferred from the gel onto a polyvinylidene fluoride membrane (PVDF, Millipore) using a Trans-Blot Turbo Transfer System (Bio-Rad). The membrane was blocked with 5% non-fat dry milk in TBST for 30 min at room temperature and incubated with primary rabbit anti-human CD9 antibody (Abcam, ab92726), rabbit anti-human syntenin antibody (Abcam, ab133267), rabbit anti-human calnexin antibody (Abcam, ab213243), or rabbit anti-human actin antibody (Abcam, ab179513), overnight at 4°C. Following incubation with HRP-conjugated goat anti-rabbit immunoglobulin G (IgG) (1:3000, Abcam, ab6721), the blot was developed using chemiluminescent reagents from Advansta. Images were captured using an Amersham Imager 600 (GE Healthcare Life Sciences).

***Untargeted lipidomics***

In brief, lipids were separated on a Waters ACQUITY PREMIER CSH C18 Column (1.7 μm, 2.1 × 100 mm), under the following chromatographic conditions: mobile phase A (acetonitrile: water = 6:4, v/v) and mobile phase B (acetonitrile: isopropanol = 1:9, v/v) at a flow rate of 300 μL/min and column oven temperature at 45°C. The gradient started with 30% of B and was held for 2 min, which was then increased to 100% of B over 23 min, the gradient was returned to 30% B over 1 min, and was finally equilibrated for 9 min. During the whole analysis, the sample was placed in an automatic injector at 10℃. Random injection sequence was used for analysis of samples to avoid the influence of signal fluctuation. MS detection was performed using a Thermo Scientific™ Q Exactive mass spectrometer mass spectrometer, equipped with an ESI ion source. Data were acquired in both positive and negative ion modes, respectively. Data-dependent acquisition methods were used for MS/MS analyses of lipidome. 10 MS2 scans were collected after each MS1 full scan. The resolution of MS1 is 70000 at m/z 200 and resolution of MS2 is 17500 at m/z 200. The ESI conditions were set at follows: Heater Temp 300°C, Sheath Gas Flow rate 45 arb, Aux Gas Flow Rate15 arb, Sweep Gas Flow Rate 1arb, spray voltage 3.0KV, Capillary Temp 350°C, S-Lens RF Level 50%, MS1 scan ranges: 200-1800. Lipidsearch 4.0 software was used for peak detection and annotation of lipids or internal standards. The main parameters were as follows: precursor tolerance 5 ppm, product tolerance 5 ppm, product ion threshold 5%.

**Supplementary Figures**

**
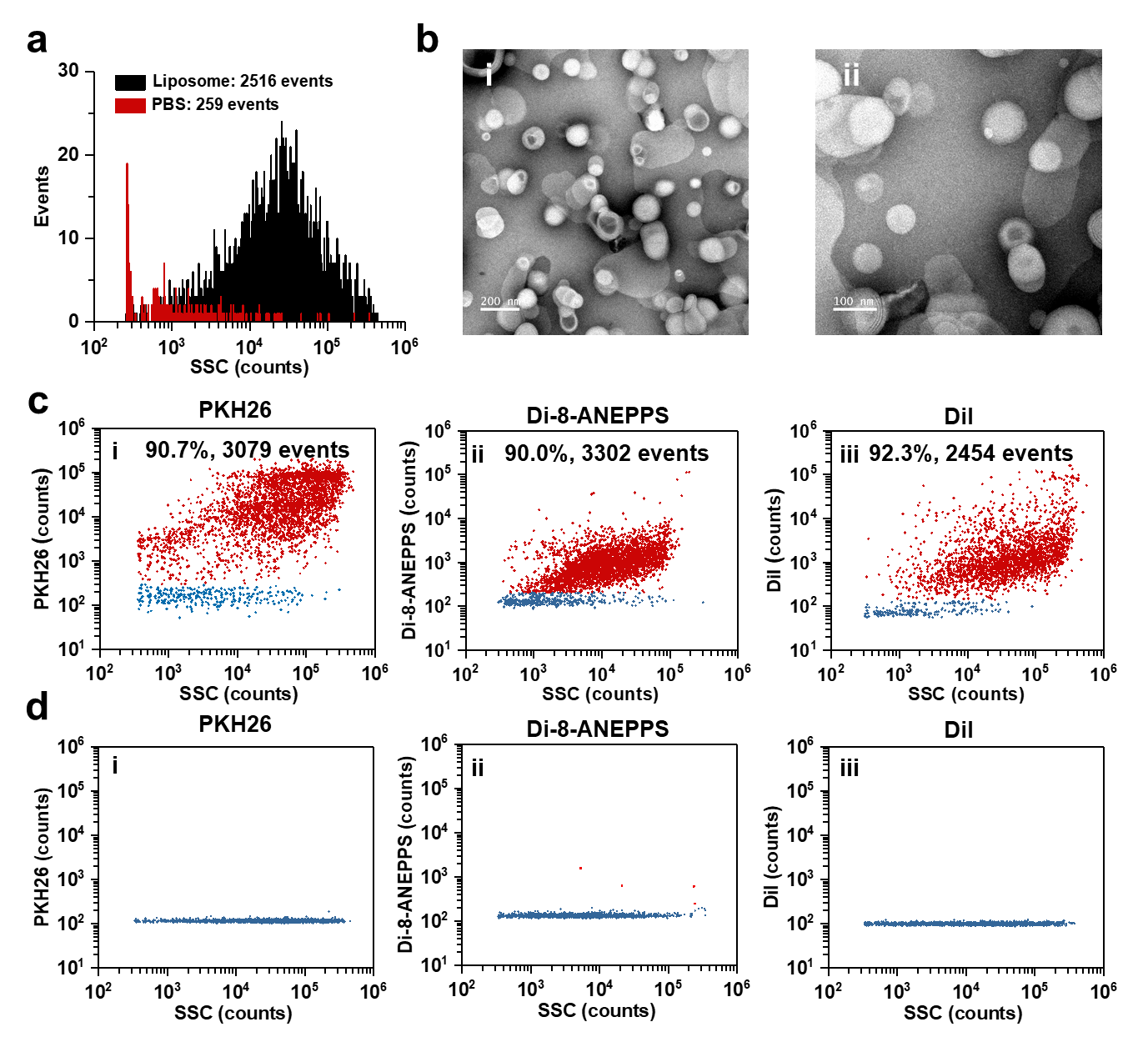
**

**Figure S1.** (a) Representative SSC burst area distribution histograms of unstained DSPC liposomes and PBS. (b) Representative TEM micrographs of synthesized DSPC liposomes with different magnifications. (c, d) Bivariate dot-plots of LMD fluorescence versus SSC for PKH26 (i), di-8-ANEPPS (ii), and DiI (iii)-labeled DSPC liposomes (c) and unlabeled DSPC liposomes (d).


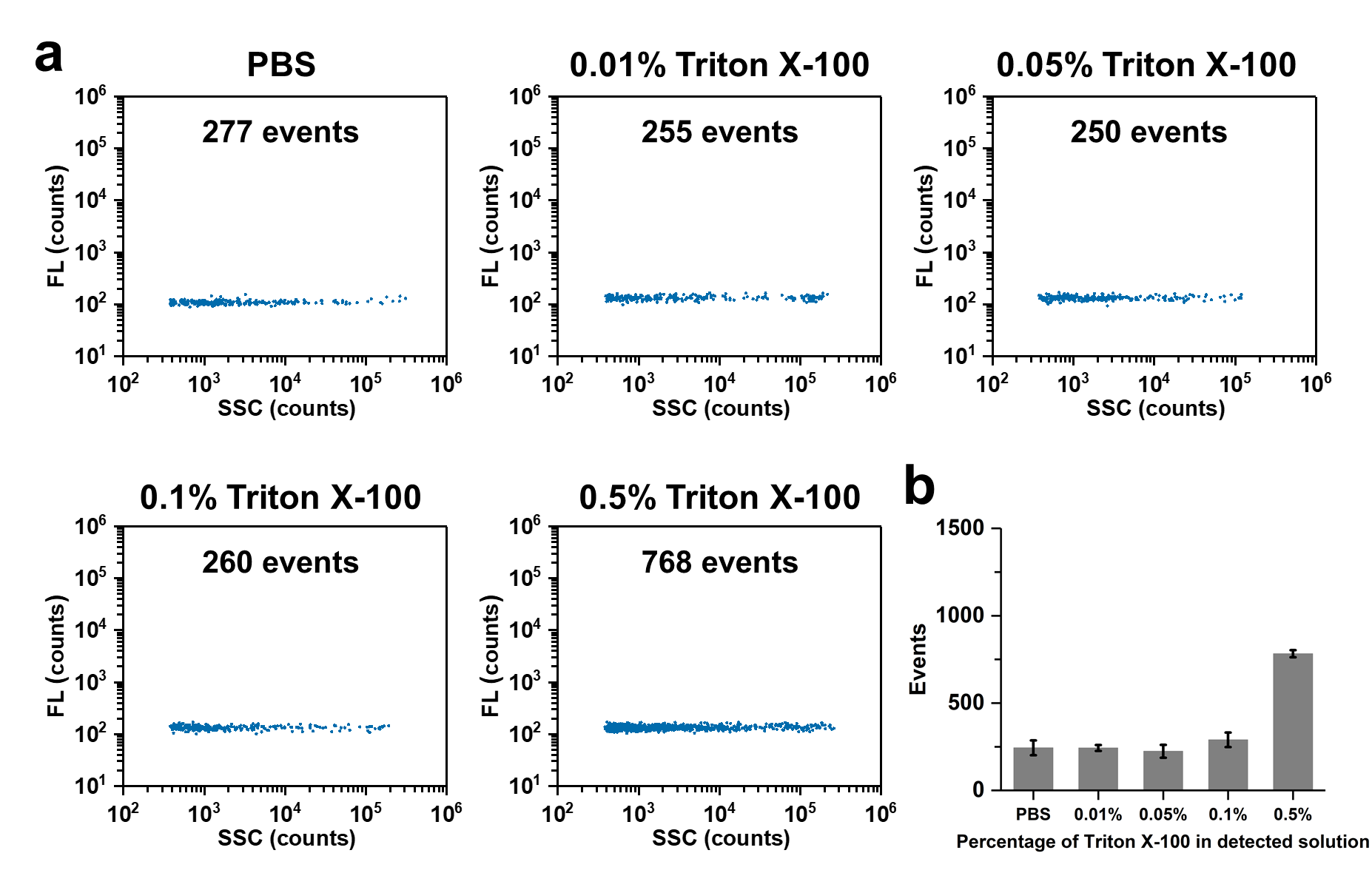


**Figure S2**. Investigation of the effect of Triton X-100 volume fraction on micelle formation in PBS without the presence of liposomes or EVs. (a, b) The bivariate dot-plots of fluorescence versus SSC (a) and the event rate measured in 1 min (mean ± s.d.) (b) for PBS containing different volume fractions of Triton X-100.

**
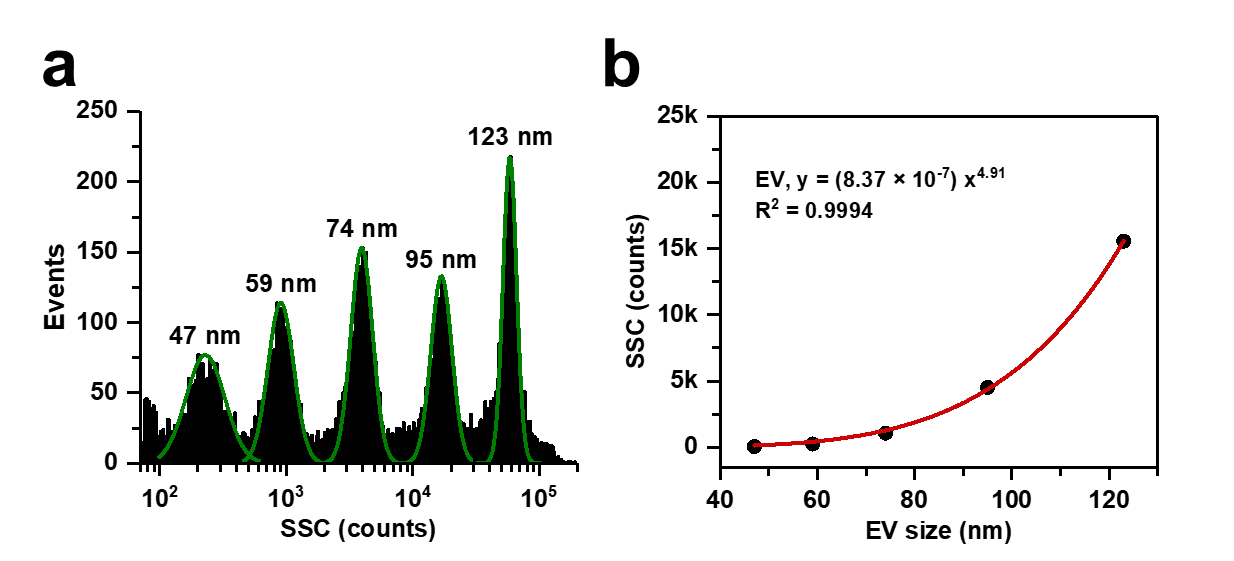
**

**Figure S3.** (a) SSC distribution histogram of a mixture of monodisperse SiNPs of five different diameters ranging between 47 and 123 nm measured on the nFCM via 532 nm laser excitation at ~10 mW, and fit to a sum of Gaussian peaks. (b) Plot of the Gaussian-fitted SSC intensity (after refractive index correction) as a function of EV size.


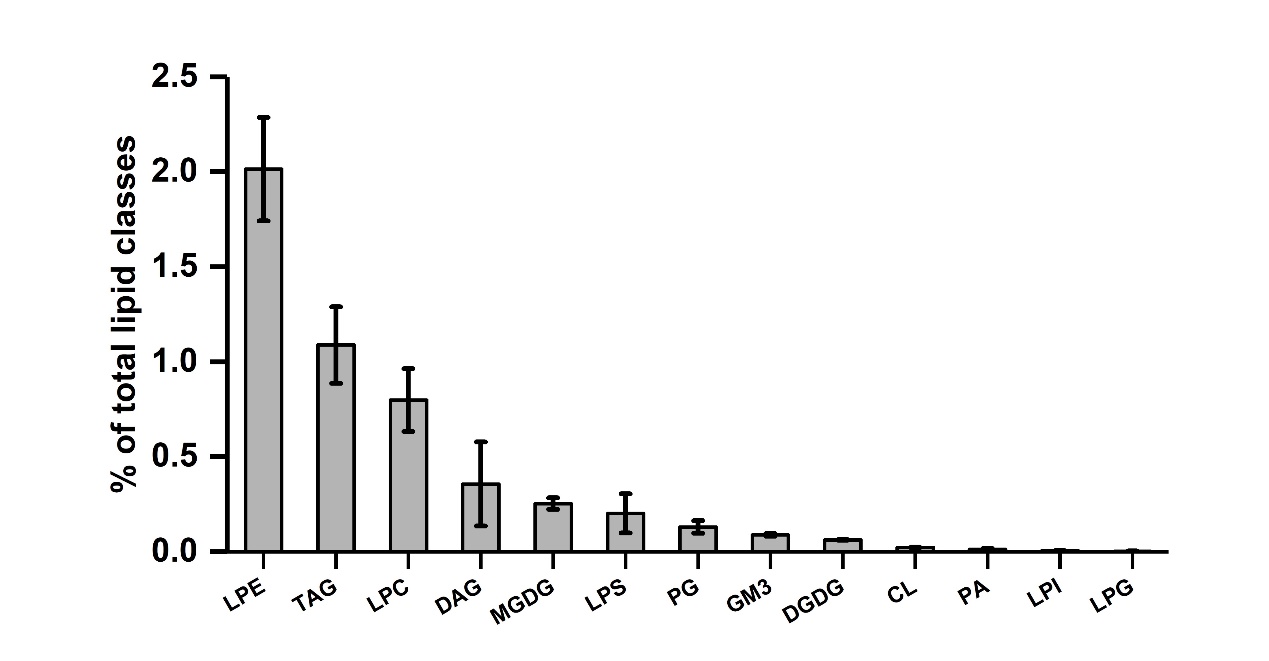


**Figure S4.** Lipid content of less-abundant lipid classes was assessed for EVs of 3 different batches. Results are expressed as the mean ± SD of the batches. LPE, lysophosphatidylethanolamine; TAG, triacylglycerol; LPC, lysophosphatidylcholine; DAG, diacylglycerol; MGDG, monogalactosyldiacylglycerol; LPS, lysophosphatidylserine; PG, phosphatidylglycerol; GM3, ganglioside-3; DGDG, digalactosyl diglyceride; CL, cardiolipin; PA, glycerophosphates; LPI, lysophosphatidylinositol; LPG, lysophosphatidylglycerol.


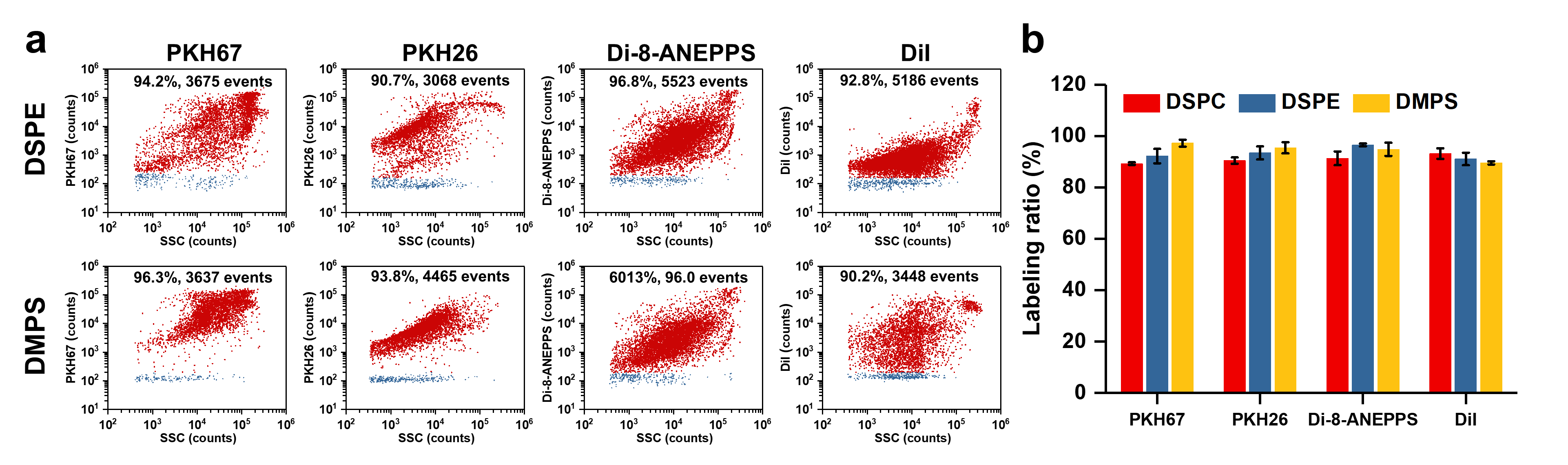


**Figure S5**. Labeling of synthesized DSPE and DMPS liposomes by LMDs. (a) Bivariate dot-plots of LMD fluorescence versus SSC for DSPE and DMPS liposome samples labeled with 4 μM PKH67, PKH26, di-8-ANEPPS, or DiI. (b) Labeling ratio comparison for DSPC, DSPE, and DMPS liposomes by all the four LMDs.

**Note:** Briefly, DSPE (1,2-distearoyl-sn-glycero-3-phosphoethanolamine) or DMPS (1,2-dimyristoyl-sn-glycero-3-phospho-L-serine) (all purchased from Avanti Polar Lipids, Inc.) were mixed with cholesterol and dissolved in chloroform at a molar ratio of 60:40 with a total lipid concentration of 100 mM. Once the lipids were thoroughly mixed, the solvent was dried on a rotary evaporator to form a lipid film and kept in vacuo for at least 3 h to remove the residual chloroform. The lipid film was hydrated in PBS solution at 65°C for 1 h followed by sequential extrusion through polycarbonate membranes with pore size of 400, 200, 100, 80, and 50 nm to obtain homogeneous particle size. After 20 cycles of extrusion with every size of the polycarbonate filters down to 50 nm, the synthesized 50 nm liposomes were stored at 4°C for further use.


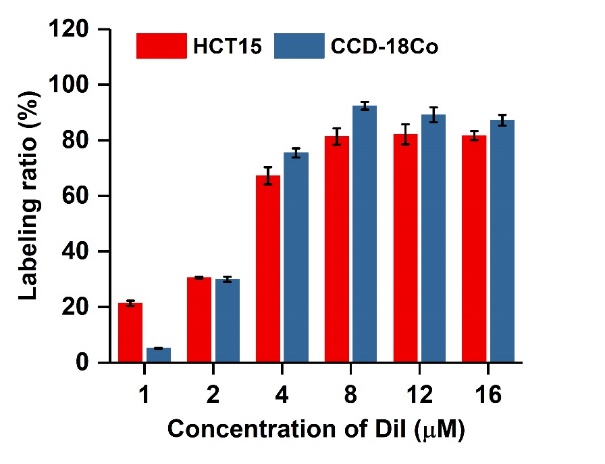


**Figure S6**. Optimization of DiI concentration for the labeling of EVs isolated from the CCCM of HCT-15 and CCD-18Co cells.

**
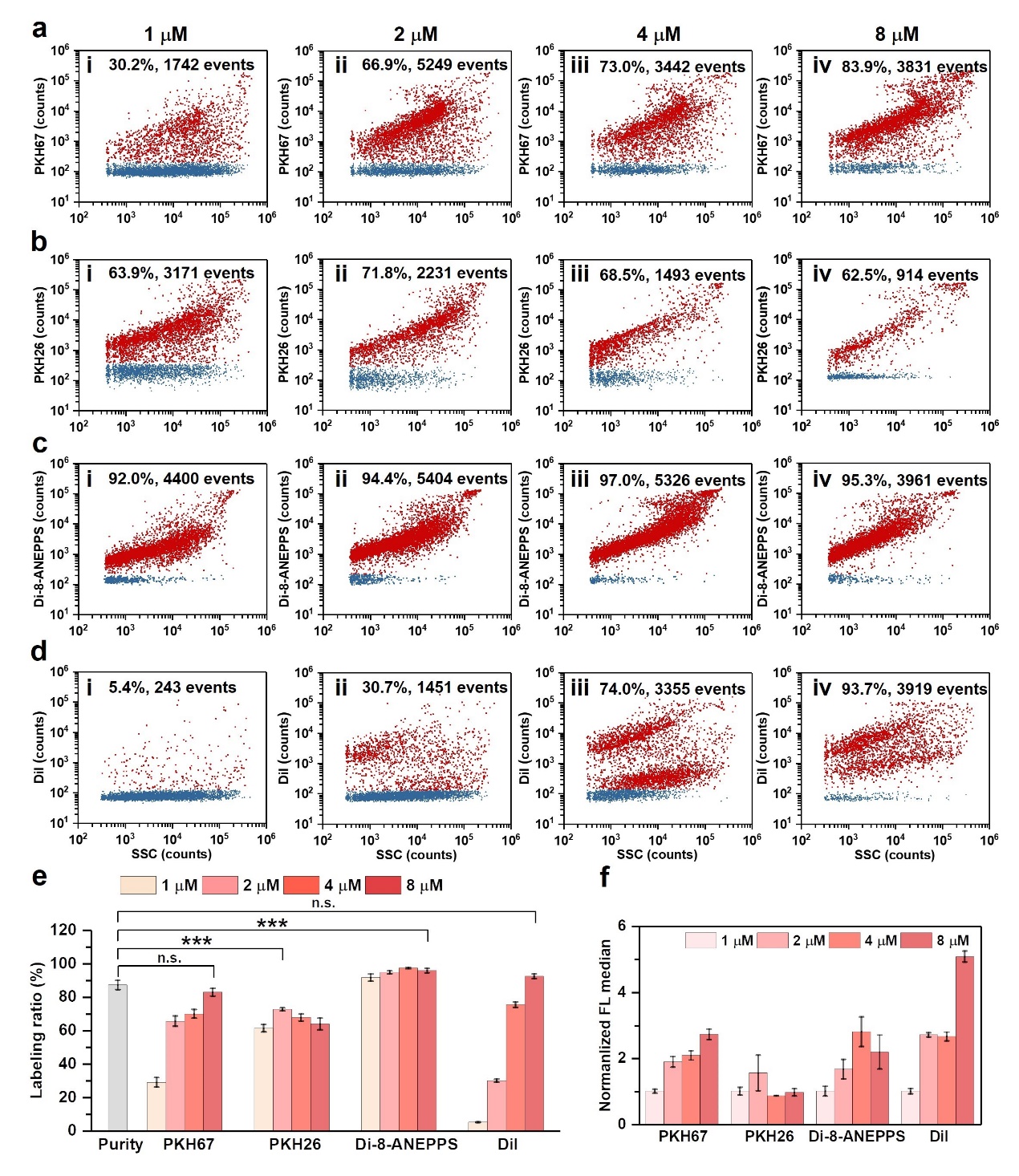
**

**Figure S7.** Analysis of membrane labeling efficiency of LMDs for EVs isolated from the CCCM of CCD-18Co cell line. (a-d) Bivariate dot-plots of FL versus SSC for PKH67 (a), PKH26 (b), di-8-ANEPPS (c), and DiI (d)-labeled EVs at 0 μM (i), 1 μM (ii), 2 μM (iii), 4 μM (iv), and 8 μM (v), respectively. (e) Three replicate measurements of EV purity by Triton X-100 assay and labeling ratio of EVs for four different LMDs at four different concentrations (mean ± s.d.). (f) Normalized median FL intensity of EVs labeled by LMDs (n = 3, mean ± s.d.). Group differences between EV purity and the highest labeling ratio of different LMDs (4 μM for PKH67, 4 μM for PKH26, 8 μM for di-8-ANEPPS, and 8 μM for DiI) were tested by one-way ANOVA analysis followed by post-hoc Bonferroni’s test for multiple comparison. P value of <0.05 was considered statistically significant. P＜0.05, *; P＜0.01, **; P＜0.001, ***.


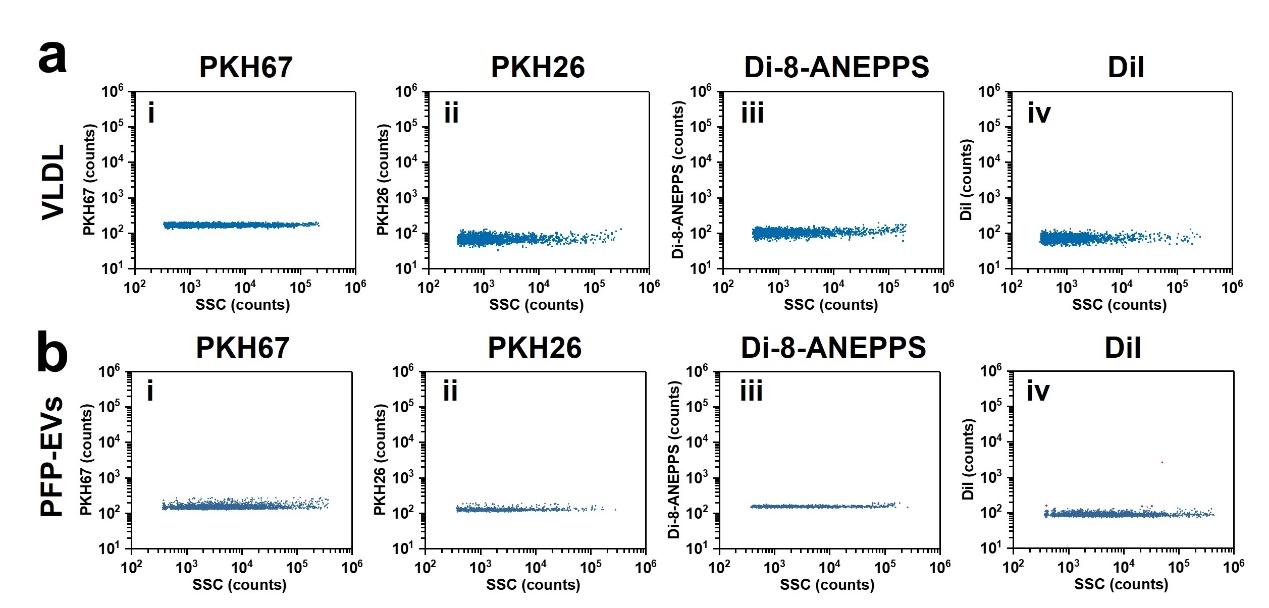


**Figure S8.** Blank control for unstained VLDL and PFP-EVs (Donor #3). (a, b) The bivariate dot-plots of LMD fluorescence versus SSC for unlabeled VLDL (a) and PFP-EVs (Donor #3) (b) at the instrument conditions used for PKH67 (i), PKH26 (ii), Di-8-ANEPPS (iii), and DiI (iv), respectively.

**
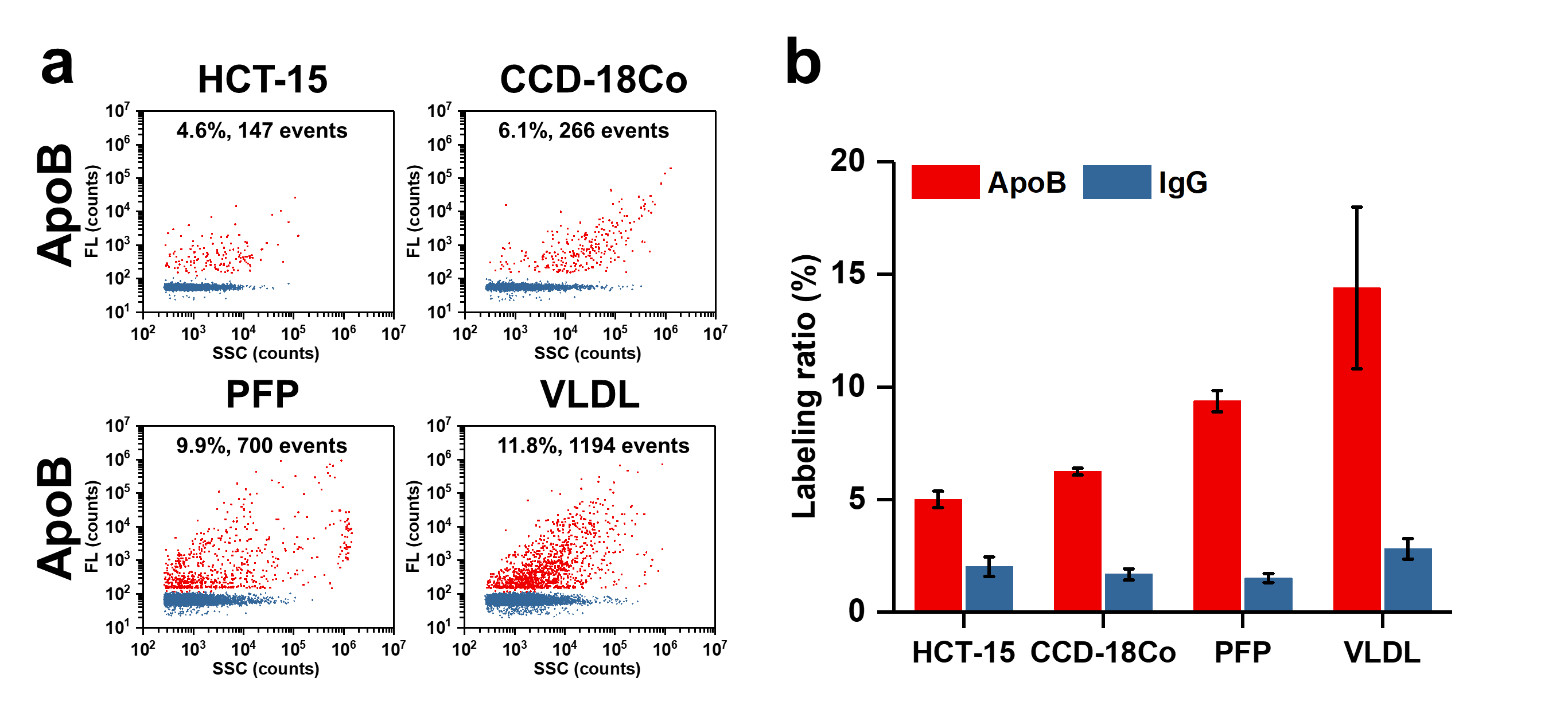
**

**Figure S9**. ApoB expression level for EVs (isolated from HCT-15, CCD-18Co, or PFP) and VLDL through antibody staining (abcam, ab139401, AF647-labeled).


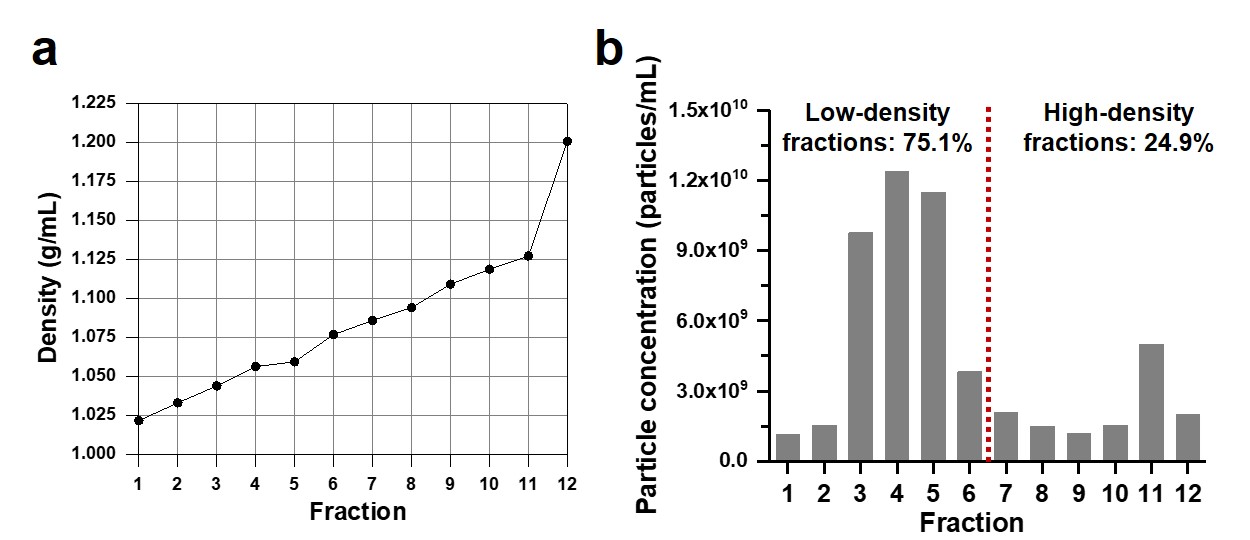


**Figure S10**. Density gradient ultracentrifugation of PFP-EVs isolated from Donor #3. (a) The measured density for each iodixanol fraction. (b) Particle concentration of each fraction.

**Note:** Density gradient ultracentrifugation was carried out for the purity assessment of PFP-EVs (Donor #3) according to the procedure reported in the literatures (*Cell* 2019, 177, 428-445; JEV 2022, 11, e12206) with minor modification (detailed protocol is described below). The low-density fractions (fraction 1-6) were identified as EV-enriched fraction and the high-density fractions (fraction 7-12) were identified as fractions enriched with non-EV particles (*Cell* 2019, 177, 428-445). As shown in Figure S9b, the particle concentrations of low-density fractions (fraction 1-6) accounted for 75.1% in total quantity, which is comparable with the purity measured by Triton X-100 treatment (Donor #3, 68.2% ± 1.1, n = 3). Thus the density gradient ultracentrifugation results validated the accuracy of using Triton X-100 lysis assay to determine the purity of PFP-EVs by nFCM.

**Protocol:** 24 mL freshly prepared PFP from Donor #3 was divided equally into 12 tubes, diluted to 12.5 mL with PBS in each tube, and centrifuged at 100,000 × g for 2 h at 4°C (Optima XE-90 ultracentrifuge with a SW 41Ti rotor, Beckman Coulter). All EV pellets were combined into a centrifuge tube and suspended in 12 mL of PBS, followed by a second ultracentrifugation at 100,000 × g for 2 h at 4°C. Afterwards, the supernatant was discarded, and the EVs were resuspended in 960 μL ice-cold PBS and mixed with 1440 μL ice-cold 60% iodixanol solution to a final 36% volume fraction and laid at the bottom of the centrifuge tube. Then 2.4 mL layers of 12%, 18%, 24%, and 30% iodixanol solution were subsequently overlaid forming a discontinuous gradient. Identical gradients without sample were generated in the same manner for later determination of fraction densities. The sample was ultracentrifuged at 100,000 × g (Optima XE-90 ultracentrifuge with a SW 41Ti rotor, Beckman Coulter) for 18 h. Fractions of 1 mL were collected from the top to the bottom, and the density of each iodixanol fraction was measured by weighting. Next, the sample of different fractions was transferred to a new tube, diluted, washed with PBS (up to 12 mL), and ultracentrifuged at 100,000 × g for 2 h (Optima XE-90 ultracentrifuge with a SW 41Ti rotor, Beckman Coulter). Afterwards, the supernatant was discarded, and the EVs were resuspended in 100 μL of PBS. The EV concentration of each fraction was measured by nFCM.

**
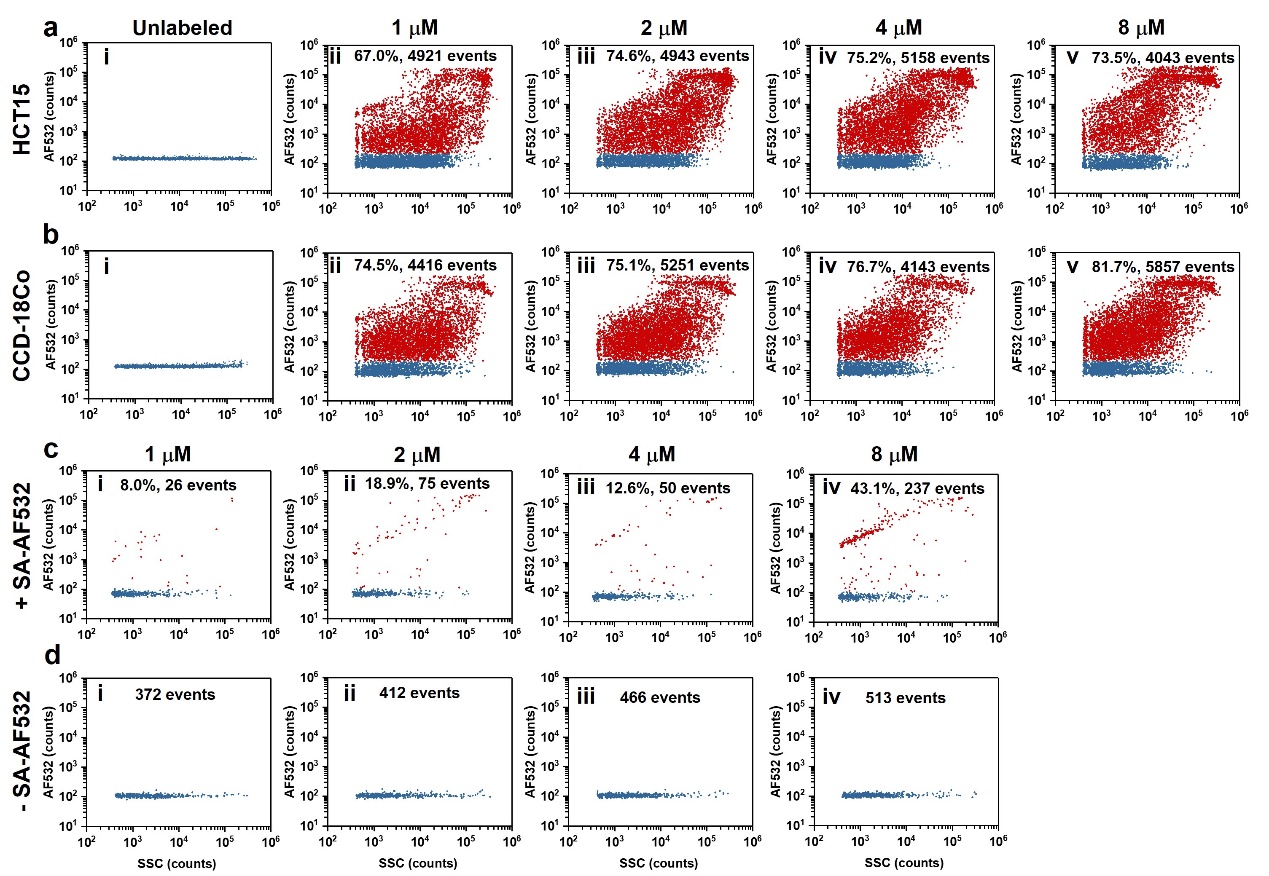
**

**Figure S11.** Characterization of DSPE-PEG_2000_-biotin labeling for EVs isolated from the CCCM. (a-b) Bivariate dot-plots of Alexa Flour-532 FL versus SSC for HCT-15 EVs (a) and CCD-18Co EVs (b) labeled by DSPE-PEG_2000_-biotin at 0 μM (i), 1 μM (ii), 2 μM (iii), 4 μM (iv), and 8 μM (v), respectively and then stained with streptavidin-AF 532. (c) Reagent controls for DSPE-PEG_2000_-biotin without the addition of EVs at 1 μM (i), 2 μM (ii), 4 μM (iii), and 8 μM (iv), respectively and then stained with streptavidin-AF 532. (d) Reagent controls for DSPE-PEG_2000_-biotin without the addition of EVs and streptavidin-AF532 at 1 μM (i), 2 μM (ii), 4 μM (iii), and 8 μM (iv), respectively.

**
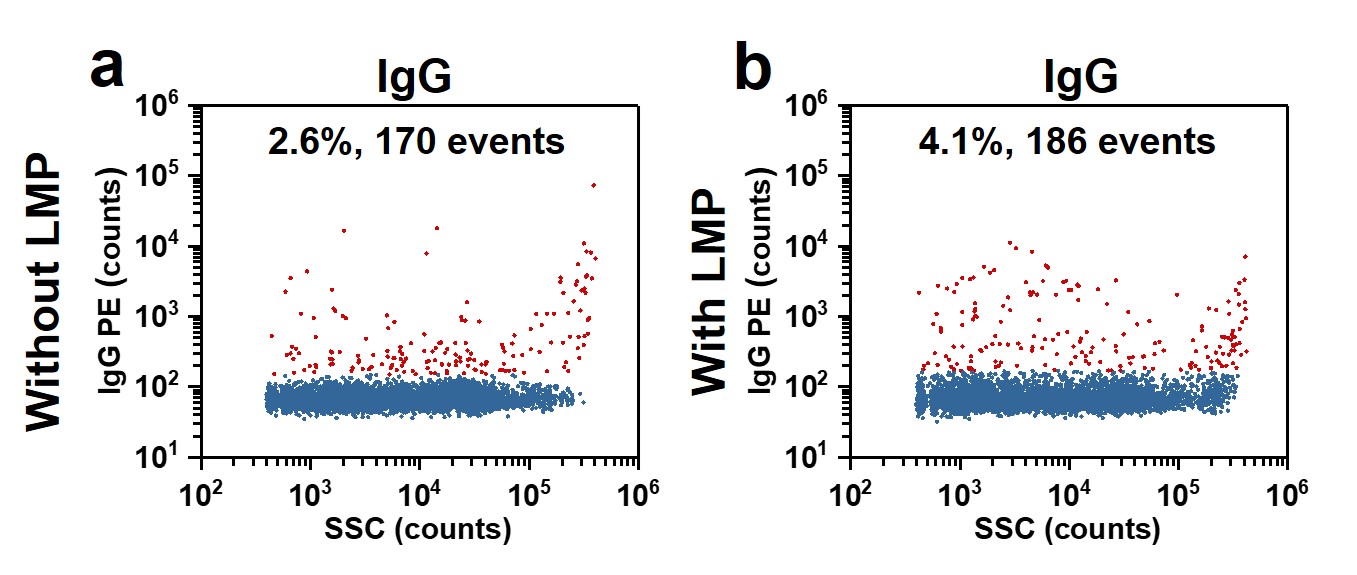
**

**Figure S12.** Bivariate dot-plots of PE-conjugated IgG FL versus SSC for EVs isolated from the CCCM of HCT-15 cells without (a) or with LMP (b) labeling.
